# 2-Hydroxychalcone−β-Cyclodextrin Conjugate with pH-Modulated Photoresponsive Binding Properties

**DOI:** 10.1021/acs.joc.2c01875

**Published:** 2022-10-15

**Authors:** Micael Paulino, Ignacio Pérez-Juste, María Magdalena Cid, José P. Da Silva, M. Manuela A. Pereira, Nuno Basílio

**Affiliations:** † Laboratório Associado para a Química Verde (LAQV), Rede de Química e Tecnologia (REQUIMTE), Departamento de Química, Faculdade de Ciências e Tecnologia, Universidade NOVA de Lisboa, 2829-516Caparica, Portugal; ‡ Facultade de Química, Edificio de Ciencias Experimentais, Universidade de Vigo, Campus Lagoas-Marcosende, 36310Vigo, Spain; § Centre of Marine Sciences (CCMAR/CIMAR LA), University of Algarve, Campus de Gambelas, 8005-139Faro, Portugal

## Abstract

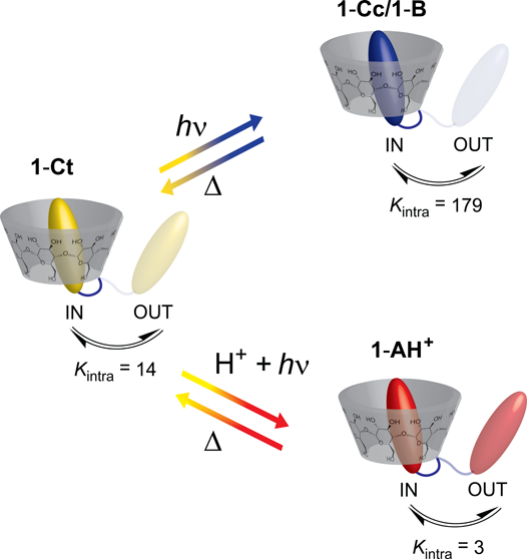

Stimuli-responsive supramolecular receptors are important building blocks for the construction of self-assembled functional materials. We report the design and synthesis of a pH- and light-responsive 2-hydroxychalcone−β-cyclodextrin conjugate (**1-Ct**) and its characterization by spectroscopic and computational methods. **1-Ct** follows the typical reaction network of *trans*-chalcone-flavylium photoswitches. Upon light irradiation, **1-Ct** can be photochemically converted into the *cis*-chalcone/hemiketal forms (**1-Cc**/**1-B**) under neutral pH conditions or to the flavylium cation (**1-AH**^**+**^) at acidic pH values. This stimuli-responsive β-cyclodextrin host, **1-Ct**, was found to form stronger intramolecular self-inclusion complexes (*K*_intra_ = 14) than **1-AH**^**+**^ (*K*_intra_ = 3) and weaker than **1-Cc**/**1-B** (overall *K*_intra_ = 179), allowing control over their stability and binding properties by combinations of pH and light stimuli.

## Introduction

The formation of host–guest binding pairs from macrocyclic hosts and small guest molecules is one of the most effective and straightforward approaches to construct self-assembled architectures in an aqueous solution.^1−6^ Within the increasing variety of water-soluble macrocycles available to supramolecular chemists, naturally occurring cyclodextrins (CDs) are among the most widely explored due to their ability to form inclusion complexes with a broad range of complementary guest molecules such as drugs, flavors, or fragrances, finding important applications in the pharmaceutical, food, and cosmetic industries.^7−11^

Even though many applications rely on native CDs, decoration of their cyclic oligosaccharide structure with different functional groups and molecules may be explored to improve their recognition abilities and expand their functional properties.^12,13^ Specifically, CD monofunctionalization, either at the primary or secondary rim, offers an appealing strategy to obtain versatile building blocks that can be applied in the construction of sophisticated self-assembled materials such as supramolecular polymers, molecular machines, soft-materials, sensors, and so forth.^14−22^ Owing to the dynamic and reversible nature of noncovalent interactions organizing and holding monomers together in these materials, CDs functionalized with stimuli-responsive molecules are often explored to control their formation, structure, properties, and, consequently, their function with chemical and physical stimuli.^14,20,23,24^ In this context, photoresponsive molecular switches are frequently preferred because light stimulus can be remotely applied with a high degree of spatiotemporal control.^25−27^ Azobenzenes are by far the most widely explored photoswitchable units due to their facile chemical modification, highly efficient and reversible *E*–*Z* photoisomerization, as well as, due to their popularity.^28,29^ Pioneering works by Ueno and co-workers have shown that CDs can be functionalized with azobenzene groups, either as capping or pendant moieties, to prepare CD-based receptors displaying photoresponsive affinity toward guest molecules.^30,31^ Since then, different photoswitches and strategies were explored to devise photoresponsive CDs.^24^ However, light-responsive CD derivatives whose binding affinity can be further controlled by orthogonal stimuli, such as pH, redox, or chemical inputs, remain less explored.^24^ Such systems offer higher-level control over the host–guest assemblies, which can be eventually explored for developing self-assembled materials with functional properties controlled according to molecular logic operations.^32−34^

**Scheme 1:**
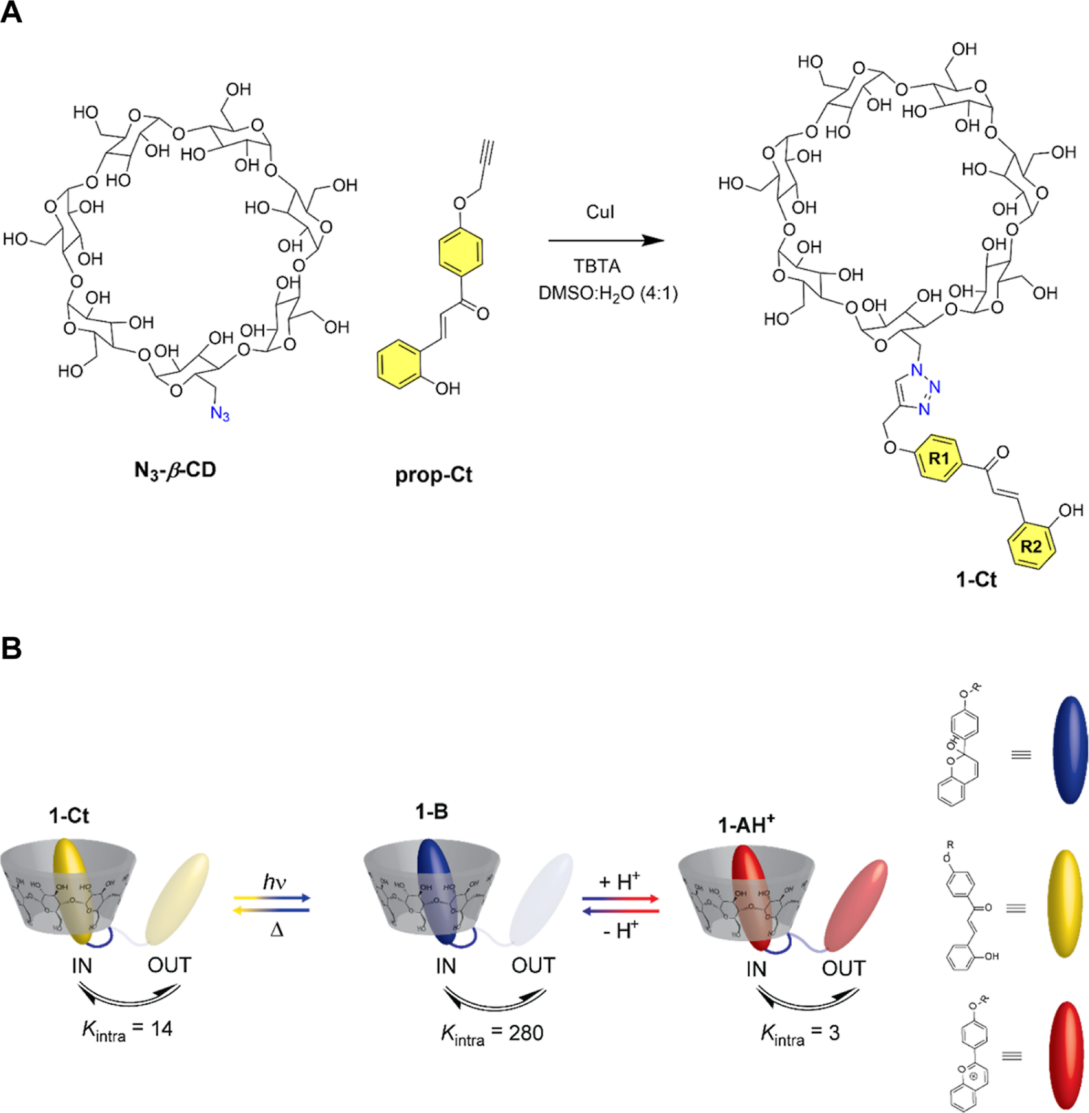
(A) Synthetic Scheme to Obtain the **1-Ct** Conjugate; (B) Control Over the Self-Inclusion Complex Affinity through the *trans*-Chalcone **1-Ct** (Moderate Affinity)–Hemiketal **1-B** (High Affinity) or **1-Ct**–Flavylium **1-AH**^+^ (Low Affinity) Interconversion Using Only Light or Light and pH Stimuli, Respectively

Flavylium salts comprise a large family of natural and synthetic dyes that have been explored to devise multistate/multiresponsive photoswitches.^35,36^ The relevant photoinduced interconversion between a hydrophobic *trans*-chalcone species and the flavylium cation under slightly acidic conditions has been shown to provide ideal conditions for the development of robust light-responsive host–guest complexes with CDs, cucurbiturils, and sulfonatocalixarenes.^37−40^ β-CD was reported to bind *trans*-chalcones with association constants (*K*) on 10^3^ to 10^4^ M^–1^ range and much lower or no affinity for the flavylium cation, allowing the dissociation of the respective host–guest complexes with light in moderately acidic conditions.^37,41−43^ In this work, we report a multistimuli-responsive β-CD derivative bearing a pendant *trans*-chalcone unit (**1-Ct**, Scheme 1A) that folds into the hydrophobic cavity of the macrocyclic ring forming a self-inclusion complex. We demonstrate that irradiation of the **1-Ct** species with UV-light at neutral pH leads to the formation of the hemiketal species (**1-B**), increasing the stability of the self-inclusion complex, while in acidic conditions, the photoinduced formation of the flavylium species (**1-AH**^**+**^) decreases its stability (Scheme 1B). The modulation of the self-inclusion complex stability with light and pH inputs enables control over the binding affinity of the β-CD toward other guest species that compete with the self-inclusion complex.

## Results and Discussion

### Structural Characterization

The attachment of functional small molecules such as dyes, photoswitches, or other stimuli-responsive molecular units to CD scaffolds can be conveniently performed using the copper-catalyzed azide–alkyne cycloaddition (CuAAC).^44^ In this work, we have successfully obtained a β-CD-*trans*-chalcone conjugate (**1-Ct**) in 75% yield after purification (Scheme 1) by coupling the monoazido β-CD (**N**_**3**_**-β-CD**), which was obtained from the native β-CD in two steps (see the Experimental Section), with a propargyl *trans*-chalcone (**prop-Ct**) using 5 mol % of CuI and 10 mol % of the Cu(I) stabilizing ligand *tris*-(benzyltriazolylmethyl)amine (TBTA)^45^ in DMSO/H_2_O.

**1-Ct** is soluble in DMSO but only slightly soluble in water precluding NMR studies in this solvent. The aromatic region of the ^1^H NMR spectrum of **1-Ct** in DMSO-*d*_6_ shows the expected set of signals corresponding to the *trans*-chalcone dye. Partial assignment of the ^1^H NMR signals was supported by COSY, ROESY, HSQC, and HMBC 2D experiments (see Figures S5–S11). In particular, the singlet at 8.23 ppm assigned to the triazole unit supports the formation of the Huisgen [3 + 2] cycloaddition product, while the pair of doublets (8.04/7.87 ppm) with *J* = 15.6 Hz confirms that the chalcone is in the *trans* configuration.

**Figure 1:**
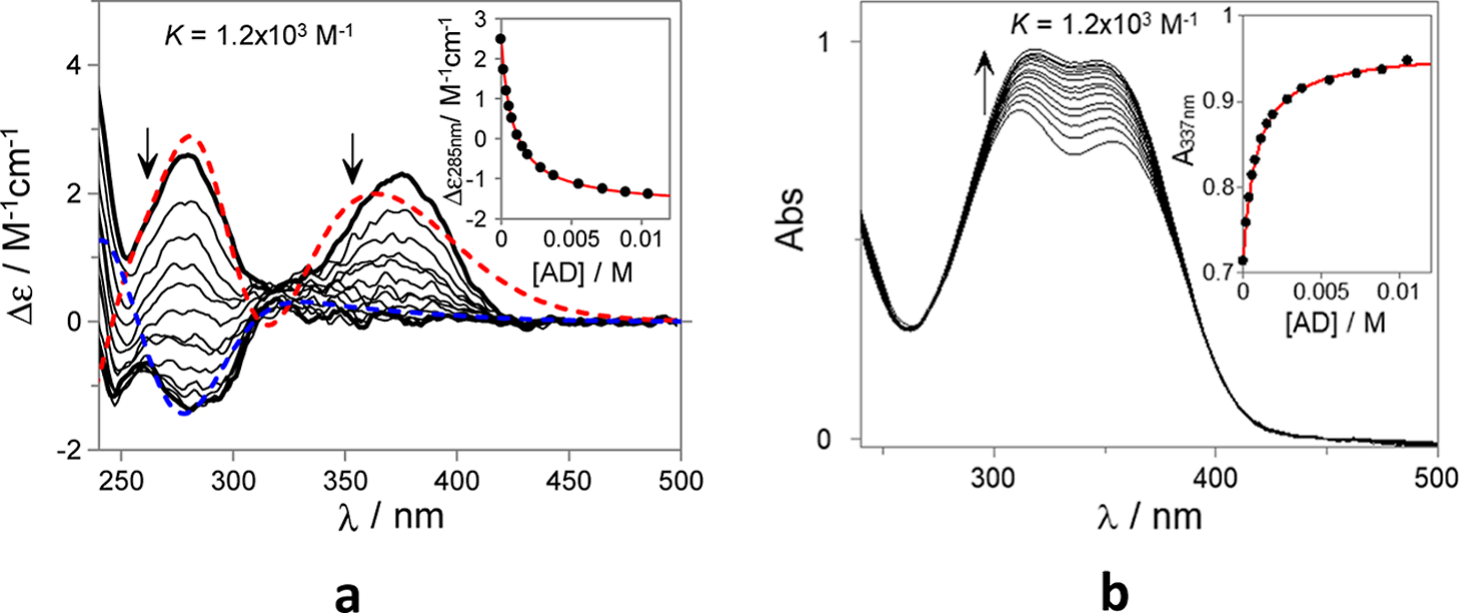
(**a**) ECD and (**b**) UV–Vis absorption spectra of **1-Ct** (35 μM 2% of DMSO in H_2_O v/v) at pH = 6 in the presence of increasing amounts of AD. The red and blue dotted lines correspond to the simulated ECD spectra of selected *trans*-chalcone conformations inside and outside the CD cavity, respectively. The simulated ECD are presented in arbitrary units.

After some screening, **1-Ct** was found to be enough soluble in 2:98 DMSO/H_2_O (v/v) for electronic circular dichroism (ECD) and ultraviolet–visible (UV–Vis) spectroscopic studies. Preliminary concentration-dependent UV–Vis spectra (see Figure S16) suggest that **1-Ct** does not aggregate (i.e., dimer or higher order supramolecular oligomers) in the concentration range employed in this work. Evidence for the self-inclusion of the *trans*-chalcone arm inside the cavity of the CD was obtained by ECD. Adamantylammonium (AD), which is a well-known complementary guest for β-CD, was used throughout this work as a competitive binder to displace the switchable arm from the cavity of the β-CD conjugate and investigate the spectroscopic properties of the unfolded conformations. As can be observed in Figure 1, the ECD spectrum, in the absence of AD, shows two bands (375 and 280 nm) that can be assigned to induced circular dichroism arising from the inclusion of the achiral chromophore inside the cavity of the CD. Upon gradual addition of AD, the lower energy ECD band disappears, while the higher energy band inverts from positive to negative, indicating the formation of a host–guest complex between AD and **1-Ct**.

**Figure 2:**
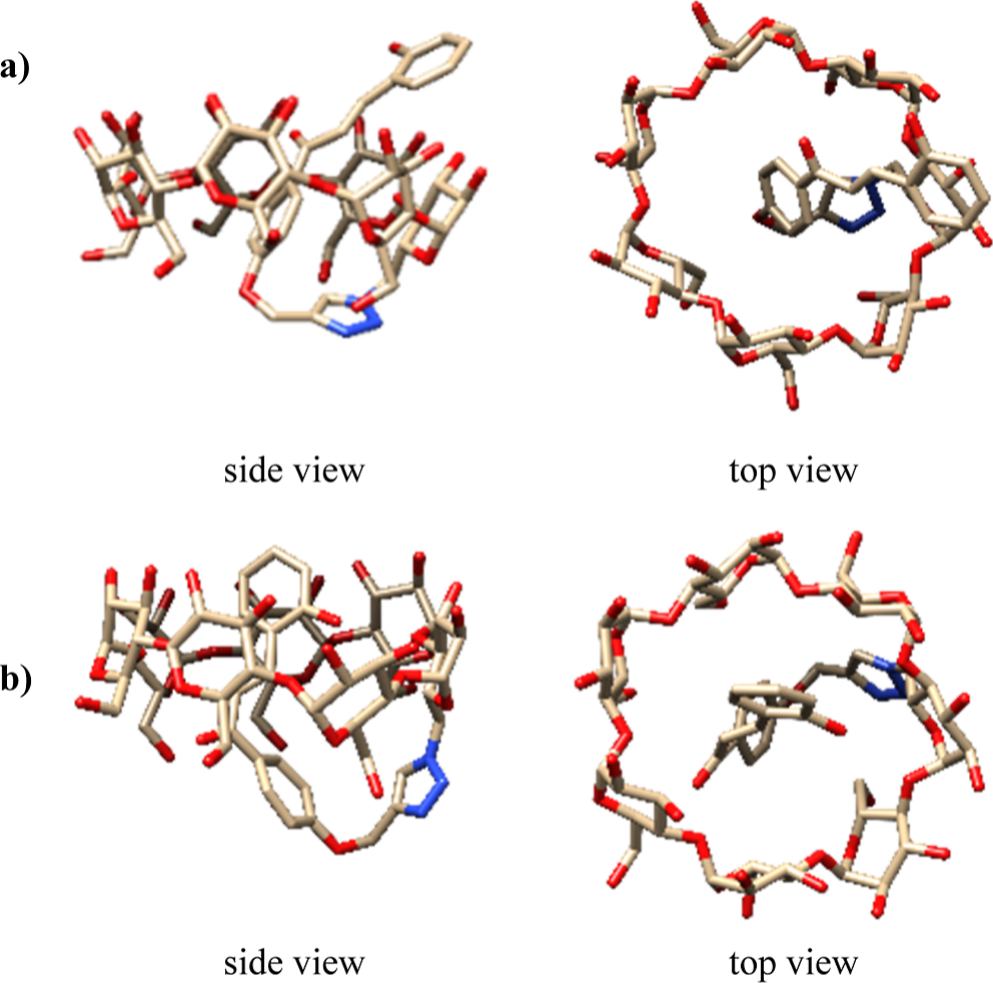
Side (left) and top views (right) of two representative conformations for **1-Ct** with either the R1 (**a**) or the R2 (**b**) phenyl ring included in the β-CD cavity. R1 corresponds to the phenyl ring directly attached to the triazole units and R2 to the remaining one. Please see Figure 1 for a clear identification of the R1 and R2 phenyl rings.

DFT calculations and molecular dynamics (MD) simulations were performed to get further insights into the structure of **1-Ct** and rationalize the ECD observations. The MD simulations evidenced the high conformational flexibility of **1-Ct** with the chalcone arm moving up and down inside the β-CD cavity (see Figure S25). The dominant conformations correspond to those where the chalcone arm is deeply buried in the β-CD cavity with the R1 ring almost parallel to the central axis and the R2 ring clearly above the large rim of the macrocycle (Figure 2a). Interestingly, for this representative structure, the chalcone unit has enough conformational flexibility to allow planar dispositions with the C=O and OH groups in a *syn*-periplanar conformation. In contrast, when the R2 ring is clearly inside the β-CD, the C=O and OH groups adopt skew orientations with angles around +100° (Figure 2b).

After clustering the MD results, the theoretical UV–Vis and ECD spectra were estimated for selected snapshots (see Figures S25 and S32). The results show that the conformation featuring the R2 ring outside of the β-CD cavity (Figure 2a) affords a theoretical ECD spectrum that agrees reasonably well with the experimental data (see Figure 1a).

The host–guest titration data (Figure 1) suggest that the chalcone arm is displaced from the β-CD cavity upon the addition of AD, leading to the appearance of an induced negative ECD band centered at 285 nm. MD simulations show that the chalcone branch presents a much larger conformational freedom outside the β-CD cavity with a significant occurrence of dispositions with the chalcone arm almost parallel to the β-CD outer wall (see Figure S26). The change in intensity observed in the ECD can be again elucidated through Kodaka rules: every electronic transition associated with the ECD spectrum is now located outside the β-CD cavity and, consequently, the intensity of the lower energy ECD bands are significantly reduced, while the negative ECD signal corresponding to the transition at 285 nm becomes the dominant one, in good agreement with the experimental ECD spectrum.

**Figure 3:**
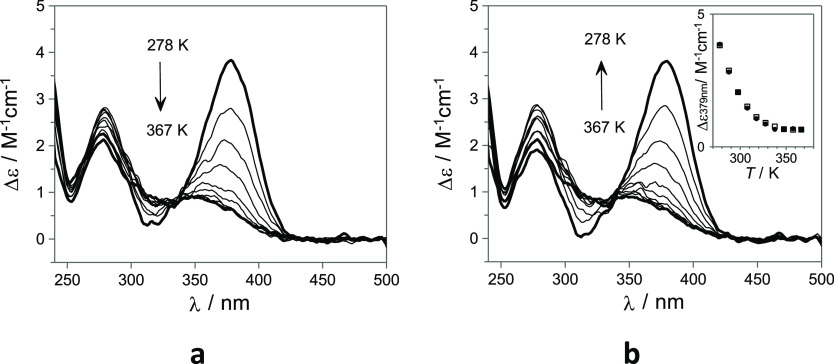
Temperature-dependent ECD spectra of **1-Ct** (35 μM 2% of DMSO in H_2_O v/v) at pH = 6. Gradual (**a**) heating and (**b**) cooling. The inset shows the ECD signal at 379 nm for heating (filled squares) and cooling (open squares).

The effect of the temperature on the self-inclusion of the *trans*-chalcone inside the cavity of β-CD was also investigated. The ECD spectra, within a temperature range from 278 to 367 K (Figure 3), show significant differences suggesting that the conformational behavior of **1-Ct** is temperature-responsive. It is worth noting that registered differences are completely reversible (see inset of Figure 3b). To assist the interpretation of the temperature-dependent ECD spectra, MD simulations were carried out at increasing temperatures from 300 to 500 K. The measurement of the distances between the β-CD center of mass (com) to the R2 ring (see Figure S27) showed that, as expected, conformational flexibility increases upon heating. Thus, the experimental spectra in Figure 3 can be related to the larger conformational mobility of the chalcone branch within the β-CD cavity. Because the centroids corresponding to the first two lower energy transitions are located near the edge of the wider rim of the β-CD cavity, these transitions occur inside and outside the cavity at higher temperatures leading to a reduction on the induced ECD. In contrast, the transitions taking place clearly inside or outside the cavity are less affected by the increased conformational mobility at higher temperatures. These findings agree well with the experimental data.

### Stimuli-Responsive Host–Guest Binding Mechanisms

**Scheme 2:**
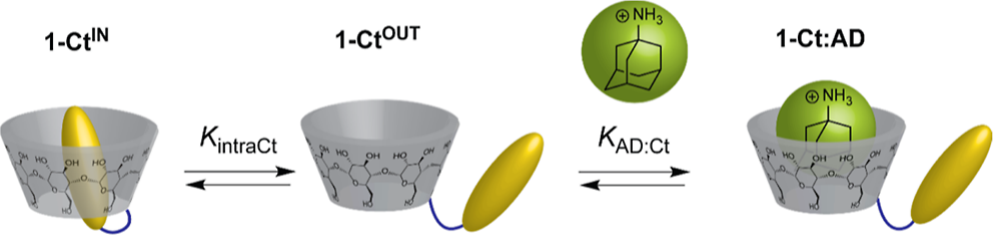
Graphical Representation of the Mechanism Proposed for the Binding of AD with **1-Ct**

The ECD and the corresponding UV–Vis titration data in Figure 1 can be fitted to obtain an apparent binding constant of *K*_app_ = 1.2 × 10^3^ M^–1^ for the formation of host–guest complexes with **1-Ct** and AD. Scheme 2 shows the proposed microscopic mechanism for the competitive displacement of the *trans*-chalcone guest from the cavity of the β-CD by AD. Considering this mechanism, it is possible to obtain the self-inclusion intramolecular equilibrium constant *K*_intra_ using eq 1 which relates *K*_app_ with the microscopic binding constants *K*_intraCt_ and *K*_AD:Ct_ (see the binding model section in the Supporting Information).^46^
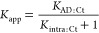
1

This approach assumes that the binding affinity of **1-Ct**^**OUT**^ toward AD is approximately equal to that of native β-CD for this guest (*K*_AD_ = 1.8 × 10^4^ M^–1^, see the Supporting Information), allowing the determination of *K*_intra:Ct_ = 14. This equilibrium constant implies that, in the absence of competitive guests (i.e., AD), the equilibrium is displaced toward the **1-Ct**^**IN**^ self-inclusion complex (ca. 93%).

The photochemistry of compound **1-Ct** was investigated at different pH values. As observed for other similar chalcone-flavylium photoswitches,^40,47^ irradiation of the *trans*-chalcone at slightly acidic/neutral pH leads to the formation of *cis*-chalcone (**1-Cc**)/hemiketal (**1-B**) mixture at the photostationary state (PSS), as confirmed by the disappearance of the UV–Vis absorption band assigned to the *trans*-species upon irradiation at 365 nm (see the Supporting Information). In presence of AD, this band does not disappear completely, suggesting the presence of *trans*-species at the PSS. The system is reversible and recovers to the *trans*-chalcone at 60 °C (see the Supporting Information) with an observed rate constant (*k*_obs_ = 6 × 10^–4^ s^–1^) that is practically independent of the presence of AD.

The composition of the PSS can be inspected by reverse pH jump experiments (see the Supporting Information), as previously described.^47^ These experiments show that in the absence of AD, the PSS is composed by 2% of **1-Ct**, 91% of **1-B**, and 7% **1-Cc**. In the presence of AD 10 mM, this distribution changes to 13% of **1-Ct**, 68% of **1-B**, and 19% **1-Cc**, suggesting that, compared with the *cis*-chalcone species, hemiketal is preferentially stabilized inside the CD cavity.

**Figure 4:**
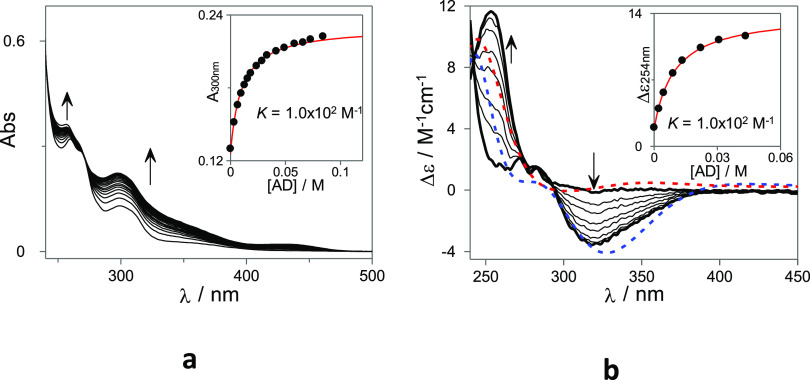
(**a**) UV–Vis absorption spectra and (**b**) ECD of **1-B**/**1-Cc** (22 μM and 63 μM for UV–Vis and ECD experiments, respectively; 2% of DMSO in H_2_O v/v) at pH = 6 in the presence of increasing amounts of AD. The red and blue dotted lines correspond to the combined simulated ECD spectra of selected *cis*-chalcone and hemiketal conformations inside and outside the CD cavity, respectively. The simulated ECDs are presented in arbitrary units.

**Scheme 3:**
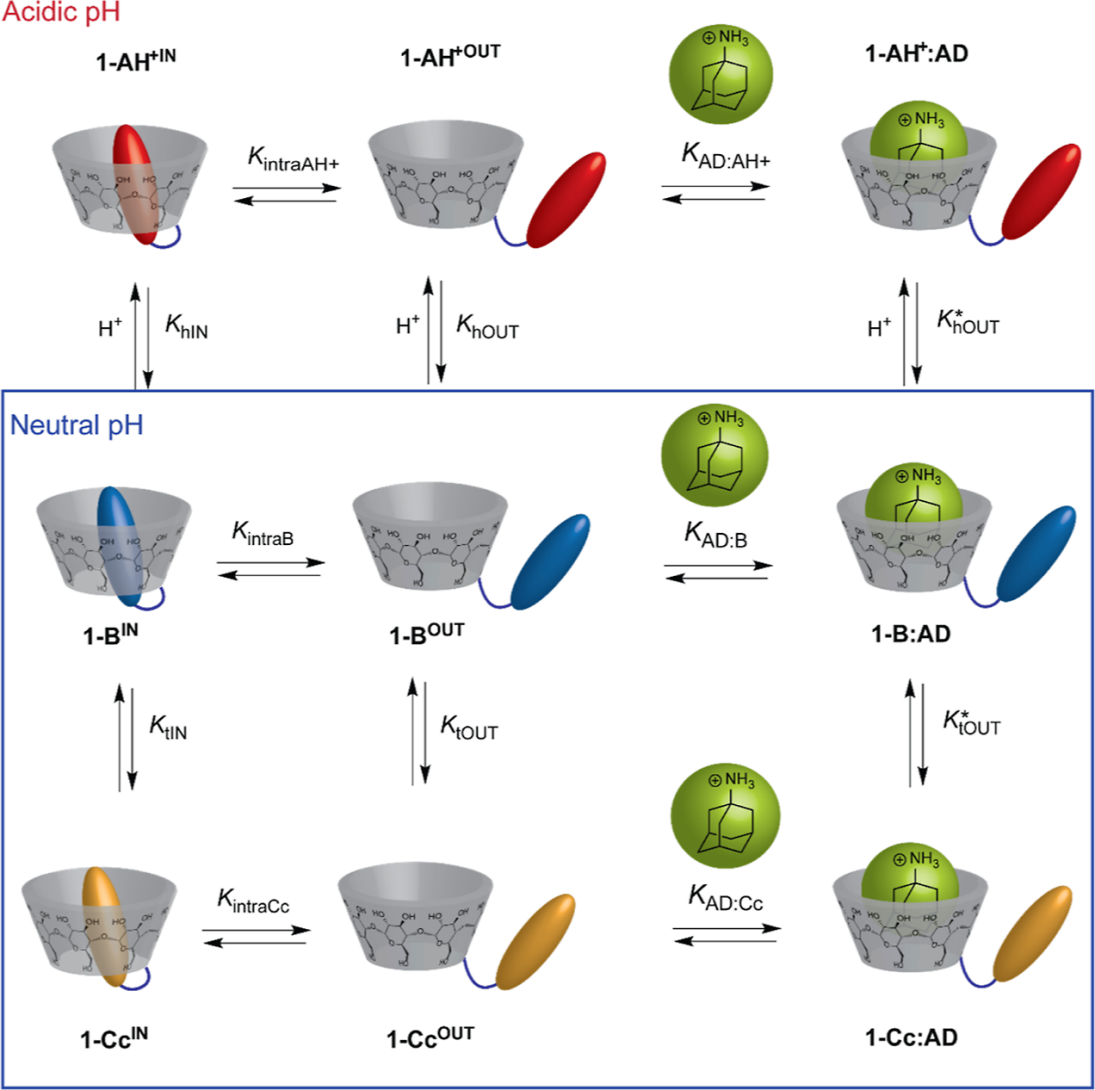
Graphical Representation of the Mechanism Proposed for the Binding of AD with Interconverting **1-B**, **1-Cc**, and **1-AH**^**+**^ Species; At Neutral pH (Blue-Framed Box), the Acidic **1-AH**^**+**^ Species is Not Thermodynamically Accessible

Because the PSS is metastable at room temperature, the formation of host–guest complexes between **1-B/1-Cc** and AD can be conveniently investigated. Figure 4 shows the UV–Vis and ECD spectral variations observed upon the addition of increasing amounts of AD to a solution containing **1-B/1-Cc** (obtained by previous irradiation of **1-Ct**). In agreement with the observations made above regarding the composition of the PSS, the UV–Vis and ECD spectral variations can be tentatively assigned to the dissociation of the **1-B** self-inclusion complex and consequent formation of a significant amount of **1-Cc**. The UV–Vis and ECD spectroscopic titration data were globally fitted to a 1:1 host–guest model with an apparent binding constant of *K*_app_ = 1.0 × 10^2^ M^–1^. This value is lower than the one observed for the **1-Ct** species (*K*_app_ = 1.2 × 10^3^ M^–1^), showing that the intramolecular self-inclusion equilibrium constants of **1-B/1-Cc** must be larger (an overall *K*_intra_ = 179 can be obtained from eq 1) than the one estimated for **1-Ct** (*K*_intra_ = 14) and therefore allows modulation of the self-inclusion conformation stability with light stimulus. In close analogy to the approach described for **1-Ct**, a host–guest complexation mechanism can be proposed to describe the association of AD with **1-B** and **1-Cc** at neutral pH (blue box in Scheme 3). It is worth noting that, under the PSS conditions, the mole fraction of **1-Ct** is only ca. 2% (see above) and hence the contribution of this species can be discarded.

In order to get further insights into the **1-B/1-Cc** system, the mole fraction of **1-B** and **1-Cc** in the presence of increasing concentrations of AD was estimated using the reverse pH jump technique (see the Supporting Information). From these data, the tautomerization constants *K*_tIN_ = [**1-Cc**]_IN_/[**1-B**]_IN_ = 0.063 and *K*_tOUT_ = [**1-Cc**]_OUT_/[**1-B**]_OUT_ = 0.69 were readily obtained from the initial and limiting mole fraction of **1-B** and **1-Cc**, respectively. These experiments provide quantitative support to the notion that the hemiketal form (**1-B**) is stabilized inside the macrocyclic cavity.

As previously described for **1-Ct**, the known equilibrium constants *K*_AD:B_ = *K*_AD:Cc_ = *K*_AD_ = 1.8 × 10^4^ M^–1^ (this approximation implies that *K*_tOUT_ = *K*_tOUT_^*^) and *K*_tIN_ and *K*_tOUT_ (obtained from reverse pH jump experiments; see above) can be combined with eqs 2 and 3 (see the Supporting Information for the demonstration) to estimate the *K*_intraB_ = 280 for **1-B** and *K*_intraCc_ = 25 for **1-Cc** showing the high stability of the **1-B** intramolecular self-inclusion complex.
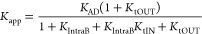
2
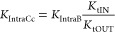
3

**Scheme 4:**
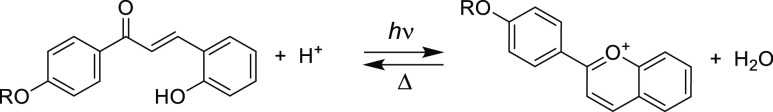
*trans*-Chalcone Photochemistry under Acidic Conditions

Under lower pH conditions, irradiation of **1-Ct** leads to the formation of the flavylium cation (Scheme 4). The primary photochemical event is the *trans*–*cis* isomerization to form a mixture of *cis*-chalcone and hemiketal, which are driven to the flavylium species under sufficiently acidic conditions, owing to the acid–base nature of the flavylium/hemiketal interconversion equilibrium. The photoinduced (λ_irr_ = 365 nm) formation of the cationic flavylium can be monitored from its absorption maximum at 434 nm (see the Supporting Information). The system is reversible, but the *trans*-chalcone recovery kinetics are much slower under more acidic conditions.

**Figure 5:**
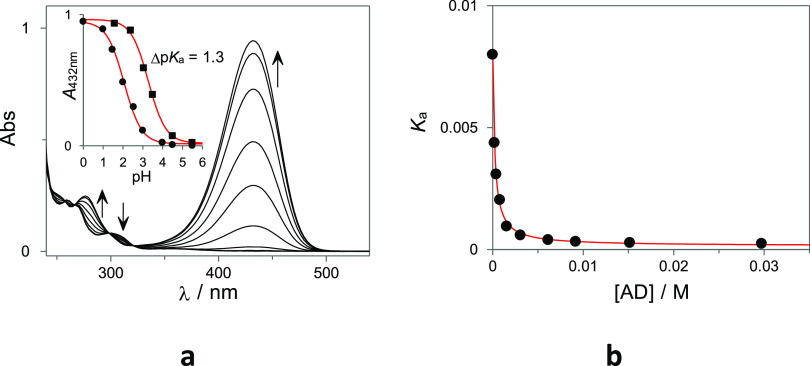
(a) UV–Vis spectra recorded at the photostationary state at different pH values obtained after irradiation of **1-Ct** (28 μM 2% of DMSO in H_2_O v/v) in 10 mM citrate buffer. Inset: absorption at 432 nm plotted against the pH in the absence (circles) and in the presence of 10 mM AD (squares). (**b**) *K*_a_ for the pseudo acid–base equilibrium established between **1-AH**^**+**^ and **1-B**/**1-Cc** (2% of DMSO in H_2_O v/v; in 10 mM citrate buffer) plotted against the AD concentration present in solution (see main text for more details).

Taking into account the acid–base nature of flavylium pH-coupled photoswitches, an apparent p*K*_a_ associated with the photoinduced formation of the flavylium cation from the *trans*-chalcone can be determined through the acquisition of the UV–Vis spectra at the photostationary state for different pH values (Figure 5a). As can be observed, the p*K*_a_ in the presence of AD increases by 1.3 p*K*_a_ units, from 2.1 to 3.4 due to the preferential stabilization of the neutral (basic) species inside the CD cavity. To estimate the self-inclusion equilibrium constant for the flavylium ion (*K*_intraAH^+^_), the p*K*_a_ for the apparent acid–base equilibrium established between the flavylium **1-AH**^**+**^ with its conjugate bases, **1-B** and **1-Cc**, was determined in the presence of increasing concentrations of AD (Figure 5b).^48^

Based on the mechanism proposed in Scheme 3 for the multistate pH-dependent equilibrium established between the **1-AH**^**+**^, **1-B**, and **1-Cc** species in the presence of AD, it is possible to derive eq 4 relating the apparent *K*_a_ for the pseudo acid–based equilibrium between **1-AH**^**+**^ with **1-B** and **1-Cc**. Under the experimental conditions, the equilibrium concentration of AD can be assumed to be approximately equal to its total AD. Using this approximation, the *K*_a_ data of Figure 5b can be fitted to eq 4 to obtain *K*_hOUT_ = 1.1 × 10^–4^ M and *K*_intraAH^+^_ = 3.0, while *K*_hIN_ = 1.0 × 10^–2^ M can be obtained from eq 5.^49^ Please note that according to this equation, the higher stability of the **1-B** self-inclusion complex with respect **1-AH**^**+**^, necessarily implies a higher hydration constant for the included dye, that is, *K*_intraB_/*K*_intraAH^+^_ = *K*_hIN_/*K*_hOUT_. The equilibrium constants defined in Schemes 2 and 3 for this multistate system are collected in Table 1. The obtained *K*_intraAH^+^_ = 3.0 shows that although this value is considerably lower than the one obtained for the neutral species, the equilibrium is displaced toward the self-inclusion complex (being 75% of **1-AH**^**+**^ in this conformation).
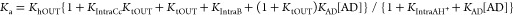
4
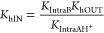
5

**Table 1:** Equilibrium Constants Obtained for the β-CD Derivative Investigated in This Work

equilibrium constant	determination method	calculated value
*K*_app_ for **1-Ct**:AD	host–guest titration (see Figure 1)	1.2 × 10^3^ M^–1^
*K*_app_ for **1-Cc**/**1-B**:AD	host–guest titration (see Figure 4)	1.0 × 10^2^ M^–1^
*K*_intraCt_	eq 1	14
*K*_intraCc_	eq 3	25
*K*_intraB_	eq 2	2.8 × 10^2^
*K*_intraAH^+^_	*K*_a_ vs [AD] data fitted with eq 4	3.0
*K*_tIN_	stopped-flow (see the Supporting Information)	0.063
*K*_tOUT_	stopped-flow (see the Supporting Information)	0.69
*K*_hIN_	eq. 5	1.0 × 10^–2^ M
*K*_hOUT_	*K*_a_ vs [AD] data fitted with eq 4	1.1 × 10^–4^ M

As described for the **1-Ct** species, a combination of ECD spectroscopy and computational studies can be applied to get further insights into the conformation of the **1-AH**^**+**^, **1-B**, and **1-Cc** forms. Under neutral pH conditions and in the absence of AD, **1-B** is the major species in solution. In fact, in the absence of AD, the observed ECD spectrum can be described if the participation of 7% of **1-Cc** and both epimers of **1-B** (see Figure 4b and S37) is considered. The **1-Cc** is characterized by two different dihedral angles that correspond with the *R* and *S* configuration of the C–OH bond in **1-B**. Interestingly, the sign of the first Cotton effect of the related **1-Cc** and **1-B** is the same both inside and outside the β-CD cavity. The analysis of the experimental and calculated ECD shows that there is an excess of the *S* epimer, inferring a higher stability of the *S*-**1-B** when compared with the *R*-counterpart. Upon the addition of AD (see Figures 4b and S38), a negative and a positive ECD bands at 320 and 250 nm, respectively, gradually appear due to the release of the hemiketal arm and its partial transformation into **1-Cc**, by ring-opening tautomerization, outside the β-CD. In these conditions, the more prominent presence of **1-Cc** in the equilibrium can be assured by the increased relevance of the band at ca 290 nm in the ECD spectrum.

**Figure 6:**
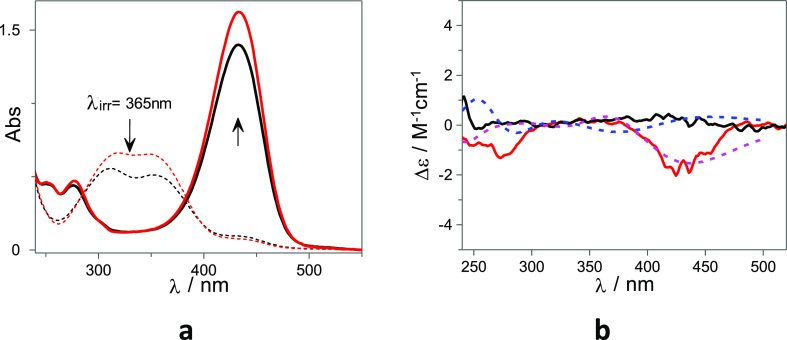
(a) UV–Vis and (b) ECD of **1-AH**^**+**^ (28 μM 2% of DMSO in H_2_O v/v) at pH = 1 in absence (black line) and in the presence of 10 mM AD (red line). The dotted lines in (a) correspond to the **1-Ct** before irradiation and in (b) to the simulated ECD spectra of selected flavylium conformations inside (blue) and outside (fuchsia) the CD cavity, respectively. The simulated ECD are presented in arbitrary units.

Figure 6 shows the UV–Vis absorption and ECD spectra of the flavylium cation, obtained upon irradiation of **1-Ct** at acidic pH, in the absence and in the presence of 10 mM AD. As can be observed, in the absence of AD, the ECD spectrum does not show any relevant band, while, on the other hand, in the presence of 10 mM AD, a negative ECD signal is observed. Related observations were reported for viologen dications appended to β-CD which are known to display a very weak affinity for the β-CD remaining outside the cavity.^50^

MD simulations indicate that the flavylium arm adopts two major conformations inside the cavity that do not interconvert during the 50 ns simulations, suggesting a hindered rotation. The calculated ECD spectra for both poses are almost mirror images (see Figure S34), which combined with an estimated of ca. 75% of self-inclusion complex (*K*_intraAH^+^_ = 3.0) may account for the absence of absorption bands in the experimental ECD spectrum (see Figure 6b). For both forms, two main electronic transitions are responsible for the ECD profiles (see Figure S35).

In the presence of AD, the flavylium arm is displaced outside the cavity of β-CD. However, as for the chalcone, despite the large mobility, there is a high occurrence of the flavylium arm adopting dispositions almost parallel to the β-CD wall (see Figure S29), regardless of the initial disposition of the flavylium fused rings. The computed ECD spectra obtained for similar configurations of the flavylium arm with different orientations of the fused rings (note the position of the oxygen ring in the insets of Figure S34) are complementary and their combination agrees well with the experimental ECD spectrum displayed in Figure 6b.

## Conclusions

This work describes the design, synthesis, and detailed characterization of a stimuli-responsive macrocyclic host based on a 2-hydroxychalcone−β-cyclodextrin conjugate (**1-Ct**). Our studies demonstrate that the pendant 2-hydroxychalcone arm is included inside the cavity of the CD forming an intramolecular self-inclusion complex. The pH-gated photoresponsive properties of 2-hydroxychalcone molecular switches allow modulation of the complex stability using combinations of these two stimuli: irradiation with UV-light (365 nm) at neutral pH triggers the transformation of the *trans*-chalcone into the *cis*-chalcone/hemiketal forms which display high affinity for the CD binding pocket. Conversely, UV-light irradiation under acidic conditions leads to the formation of the flavylium cation resulting in a lower-stability intramolecular complex. We envisage that the stimuli-responsive properties observed for this 2-hydroxychalcone−β-cyclodextrin conjugate opens possibilities to explore this and analogue systems in the construction of switchable functional materials such as supramolecular polymers, hydrogels, and molecular muscles.

## Experimental Section

### Materials

All commercially available reagents and solvents were used as received, unless otherwise stated. The propargylated *trans*-chalcone (**prop-Ct**) was available from previous studies.^51^

### General Methods

The pH of the solutions, prepared with milliQ water, was adjusted with HCl or NaOH and measured with a Crison basic 20+ pH meter. UV/vis absorption spectra were recorded using a Varian Cary 100 Bio or a Varian Cary 5000 spectrophotometer in quartz or disposable plastic cuvettes with 10 mm optical path. Circular dichroism absorption and UV–Vis spectra were recorded on a Chirascan qCD spectrometer equipped with a CS/JS Recirculating Cooler. The slower kinetic experiments were performed in a conventional spectrophotometer and the faster ones (that reach the equilibrium in less than a few minutes) were instead monitored in an Applied Photophysics SX20 stopped-flow spectrometer provided with a PDA.1/UV photodiode array detector. Continuous irradiation experiments were conducted in a SPEX Fluorolog 1681 0.22 m fluorimeter (equipped with a 150 W Xe lamp) or in a custom photochemical reactor equipped with a 200 W Hg–Xe lamp and using bandpass or cutoff filters to isolate desired wavelengths. NMR spectra were recorded using a Bruker Avance III operating at 400 MHz (^1^H) or 101 MHz (^13^C) and a Bruker NEO 500 operating at 500 MHz (^1^H) or 126 MHz (^13^C). Infrared spectra were recorded using a PerkinElmer Spectrum Two in the ATR mode. High-resolution mass spectra were recorded in the negative mode using an Orbitrap Elite mass spectrometer (Thermo Scientific), equipped with a heated electrospray ionization source (HESI-II). Solutions were infused at 5 μL/min, the spray voltage was 3.0 kV and heater temperature 45 °C.

### Titration Experiments

The spectroscopic titrations were conducted as follows: a stock solution of **1-Ct** at the desired concentration was initially prepared in DMSO. An aliquot of this solution (50 μL) was then diluted in a plastic or quartz cuvette (1 cm optical path) containing 2450 μL of H_2_O (2:98 of DMSO/H_2_O). A second solution containing exactly the same **1-Ct** concentration, volume fraction of DMSO, and a large excess of AD chloride was also prepared. The pH of both solutions was checked in a Crison basic 20+ pH meter and corrected, if necessary, to pH = 6.0 ± 0.1 using small aliquots (1–2 μL) of NaOH or HCl. Then, the titrations were carried out by adding small aliquots of the second solution of the first and measuring the UV–Vis or ECD spectra after each addition. This ensures that the concentration of **1-Ct** is kept constant while changing the AD concentration.

For the titrations carried out at the metastable state, **1-Ct** solutions were first irradiated at 365 nm in the absence of AD to generate metastable solutions at pH = 6.0 ± 0.1 containing only **1-B** and **1-Cc**, that were immediately used to perform the respective titrations. It is important to note that when **1-Ct** is irradiated in the presence of AD, there is a significant fraction of **1-Ct** at the PSS and the kinetics of the thermal back reaction from **1-B**/**1-Cc** to **1-Ct** are slow enough at room temperature to perform the experiments without significant interferences form this process. Then, the UV–Vis and ECD host–guest titrations were carried out as described above.

To determine the apparent p*K*_a_ of the **1-AH**^**+**^**:1-B**/**1-Cc** pseudo acid–base system in the absence and in the presence of increasing amounts of AD, solutions of **1-B**/**1-Cc** at different pH values were generated photochemically (as described above). Then, for each pH value, increasing amounts of AD were added to the solution and the UV–Vis spectra recorded after each addition. Due to the acid–base nature of the **AH**^**+**^**:1-B**/**1-Cc** equilibrium, these experiments were carried out in the presence of a citrate buffer (10 mM) to keep the pH constant throughout the addition of AD (checked at the beginning and at the end of the titration).

## Computational Studies

The stability of different geometries was explored by DFT calculations performed at the M062X/6-311+G* level using Gaussian.^52^ For this purpose, gas-phase geometrical optimizations followed by the calculation of vibrational frequencies for characterizing the structures as energy minima have been performed. MD simulations were performed using GROMACS 2020.3.^53^ Initial structures for systems containing the *trans*-chalcone arm inside and outside the CD cavity were built employing the coordinates for the CD in 3M3R (PDB code) and the DFT optimized structures for the *trans*-chalcone. The CHARMM36 force field,^54,55^ which has been proven to provide good results for CDs in aqueous solution,^56^ was employed to describe the CD moiety, and initial parameters for the *trans*-chalcone were obtained with the CGenFF program.^55,57^ Further refinement of the proposed parameters with larger penalties was carried out using the ffTK tool.^58^ The CD-chalcone system was solvated with water modeled with the TIP3P model in cubic boxes with a side-length of 35 Å.^59^ These structures were energy minimized in vacuum with the positions of the heavy atoms restrained with 1000 kJ mol^–1^ nm^–2^, and followed by NPT and NVT equilibration simulation of 500 ps with restraints on the heavy atoms of CD (1000 kJ mol^–1^ nm^–2^). Production run for 50 ns using a 2 fs time step and saving configurations each 10 ps. The temperature was kept constant at 300 K with the Nosé–Hoover thermostat using a coupling constant of 1 ps. The pressure was set to 1 bar with the Parrinello–Rahman barostat by isotropic coupling using a coupling constant of 5 ps. Electronic transitions within the 250–500 nm interval have been analyzed by means of natural orbital transitions orbitals (NTOs)^60^ to simplify the analysis because none of the electronic transitions can be assigned to a single electronic transition between molecular orbitals. Employing Multiwfn software,^61^ we have also obtained the centroids of the NTOs, which were used to analyze the charge transfer during electron excitation.

### Synthesis of Mono-6-deoxy-6-(*p*-toluenesulfonyl)-β-cyclodextrin (**Ts-β-CD**)^62^

A suspension of β-CD (10 g; 8.81 mmol) in water (225 mL) was heated to 65 °C until total solubilization and then cooled to room temperature. *N*-tosylimidazole (7.85 g; 32.32 mmol) was added, and the reaction was stirred at room temperature for 2 h. A solution of NaOH (4.5 g; 12.77 mmol) in water (12.5 mL) was added and stirred for 20 min. The mixture was vacuum-filtered and ammonium chloride (12.12 g; 226.60 mmol) was added to the liquid fraction, resulting in the formation of a white precipitate. The liquid fraction was concentrated by blowing a stream of air at its surface overnight. The resulting solid was vacuum-filtered and washed with ice-cold water and acetone and dried under vacuum. The desired product was obtained as a white solid (3.62 g; 2.81 mmol; 32%).

IR (neat) *ν* (cm^–1^): 3271; 2929; 1641; 1451; 1360; 1023.

^1^H NMR (500 MHz, DMSO-*d*_6_): δ 7.75 (d, *J* = 7.9 Hz, 2H), 7.43 (d, *J* = 8.1 Hz, 2H), 5.84–5.59 (m, 14H), 4.89–4.72 (m, 7H), 4.55–4.39 (m, 5H), 4.38–4.28 (m, 2H), 4.24–4.15 (m, 1H), 3.76–3.17 (m, 40H, overlapping with H_2_O/HDO), 2.43 (s, 3H) ppm.

^13^C{^1^H} NMR (126 MHz, DMSO-*d*_6_): δ 145.4, 145.1, 138.7, 132.9, 130.3, 128.7, 127.9, 125.9, 102.6, 102.3, 102.2, 102.2, 101.6, 82.0, 81.9, 81.8, 81.7, 81.4, 81.1, 73.4, 73.4, 73.1, 73.1, 72.7, 72.6, 72.5, 72.4, 72.4, 72.2, 70.1, 69.3, 60.3, 60.2, 59.9, 59.6, 21.6 ppm.

### Synthesis of Mono-6-deoxy-6-azido-β-cyclodextrin (**N**_**3**_**-β-CD**)^62^

To a suspension of **Ts-β-CD** (3.62 g; 2.81 mmol) in water (80 mL), sodium azide (2.17 g; 33.38 mmol) was added. The mixture was refluxed for 4 days, cooled to room temperature, and Amberlite IR-900 was added and stirred for 1 h. The resin was removed, and the liquid phase was concentrated, forming a white solid that was vacuum-filtered. The solid was washed with ice-cold water and acetone and dried under vacuum. The product was obtained as a white solid (2.18 g; 1.88 mmol; 67%).

IR (neat) *ν* (cm^–1^): 3307; 2927; 2106; 1021.

^1^H NMR (400 MHz, DMSO-*d*_6_): δ 5.82–5.59 (m, 14H), 4.93–4.75 (m, 7H), 4.59–4.38 (m, 6H), 3.81–3.18 (m, 42H, overlapping with H_2_O/HDO) ppm.

^13^C{^1^H} NMR (101 MHz, DMSO-*d*_6_): δ 102.3, 102.1, 102.0, 101.6, 83.0, 81.9, 81.6, 81.6, 81.4, 73.1, 73.0, 72.9, 72.8, 72.4, 72.2, 72.1, 72.1, 72.0, 70.2, 60.2, 59.9, 59.8, 51.1 ppm.

### Synthesis of Mono-6-deoxy-6-((*E*)-1*H*-1,2,3-triazolyl)methoxy)phenyl)-3-(2-hydroxyphenyl)propenone*-*β-cyclodextrin (**1-Ct**)

A solution of **N**_**3**_**-β-CD** (75 mg; 64.66 μmol), **prop-Ct** (20 mg; 71.86 μmol), and TBTA (8 mg; 15.08 μmol) in a mixture of DMSO/H_2_O (4:1) was bubbled with N_2_ for 5 min. Cuprous iodide (2 mg; 10.50 μmol) was added and the reaction mixture was stirred at 60 °C overnight in an inert atmosphere. Then, the mixture was cooled to room temperature and CupriSorb resin was added. After stirring at room temperature for 2 h, the reaction mixture was filtered and poured into ice water. The resulting yellow precipitate was collected by centrifugation. The solid was washed with water, acetone, and diethyl ether and **1-Ct** was obtained as a yellow solid (70 mg; 48.67 μmol; 75%).

IR (neat) *ν* (cm^–1^): 3360; 3001; 2915; 1654; 1600; 1015.

^1^H NMR (500 MHz, DMSO-*d*_6_): δ 10.24 (s, 1H), 8.22 (s, 1H), 8.12 (d, *J* = 8.5 Hz, 2H), 8.02 (d, *J* = 15.7 Hz, 1H), 7.89–7.82 (m, 2H), 7.26 (t, *J* = 7.6 Hz, 1H), 7.20 (d, *J* = 8.6 Hz, 2H), 6.93 (d, *J* = 8.1 Hz, 1H), 6.87 (t, *J* = 7.6 Hz, 1H), 5.91 (s, 1H), 5.84–5.60 (m, 13H), 5.22 (s, 2H), 5.08–5.02 (m, 1H), 4.99–4.89 (m, 1H), 4.88–4.78 (m, 5H), 4.79–4.75 (m, 1H), 4.65–4.57 (m, 1H), 4.57–4.44 (m, 5H), 4.06–3.94 (m, 1H), 3.81–3.19 (m, 37 H, overlapping with H_2_O/HDO), 3.17–3.08 (m, 1H), 2.94–2.82 (m, 1H) ppm.

^13^C{^1^H} NMR (126 MHz, DMSO-*d*_6_): δ 187.7, 162.0, 157.1, 142.1, 138.7, 131.9, 131.0, 130.8, 128.6, 125.7, 121.6, 120.8, 119.5, 116.2, 114.7, 102.3, 102.1, 101.9, 101.3, 83.5, 82.1, 81.7, 81.6, 81.4, 81.0, 73.3, 73.2, 73.0, 72.7, 72.5, 72.4, 72.1, 71.8, 70.1, 61.3, 60.2, 60.0, 59.9, 59.0, 50.5 ppm.

HRMS-ESI (negative mode) *m*/*z*: calculated for (C_60_H_82_N_3_O_37_) [M – H]^−^: 1436.4633; found, 1436.4663.

## Supporting Information Available

Additional spectroscopic data and experiments, comprehensive computational studies, and detailed mathematical models (PDF)
