# Supplementary material for: 2-Hydroxychalcone−β-Cyclodextrin Conjugate with pH-Modulated Photoresponsive Binding Properties
Source: J Org Chem. 2022 Oct 15;87(21):14422–32. doi: 10.1021/acs.joc.2c01875 (PMC9776619; doi:10.1021/acs.joc.2c01875)
Supplement: Supplementary file 1 — jo2c01875_si_001.pdf [file jo2c01875_si_001.pdf]

Supporting Information

A 2-Hydroxychalcone-*β*-Cyclodextrin Conjugate With pH-Modulated Photoresponsive Binding Properties

Micael Paulino,[a] Ignacio Pérez-Juste,[b] María Magdalena Cid,*[b] José P. Da Silva,[c] M. Manuela A. Pereira,*[a] Nuno Basílio*[a]

[a]Laboratório Associado para a Química Verde (LAQV), Rede de Química e Tecnologia (REQUIMTE), Departamento de Química, Faculdade de Ciências e Tecnologia, Universidade NOVA de Lisboa, 2829-516 Caparica, Portugal. E-mail: manuela.pereira@fct.unl.pt ; E-mail: nuno.basilio@fct.unl.pt

[b]Facultade de Química, Edificio de Ciencias Experimentais, Campus Lagoas-Marcosende, Vigo, 36310 Spain. E-mail: mcid@uvigo.es

[c]Centre of Marine Sciences (CCMAR/CIMAR LA), University of Algarve, Campus de Gambelas, 8005-139 Faro, Portugal.

Table of Contents

1. NMR, FTIR and HRMS data S3

2. Supplementary spectroscopic studies S11

3. Computational studies S19

4. Host-guest binding models S41

5. References S45

1. NMR, FTIR and HRMS data

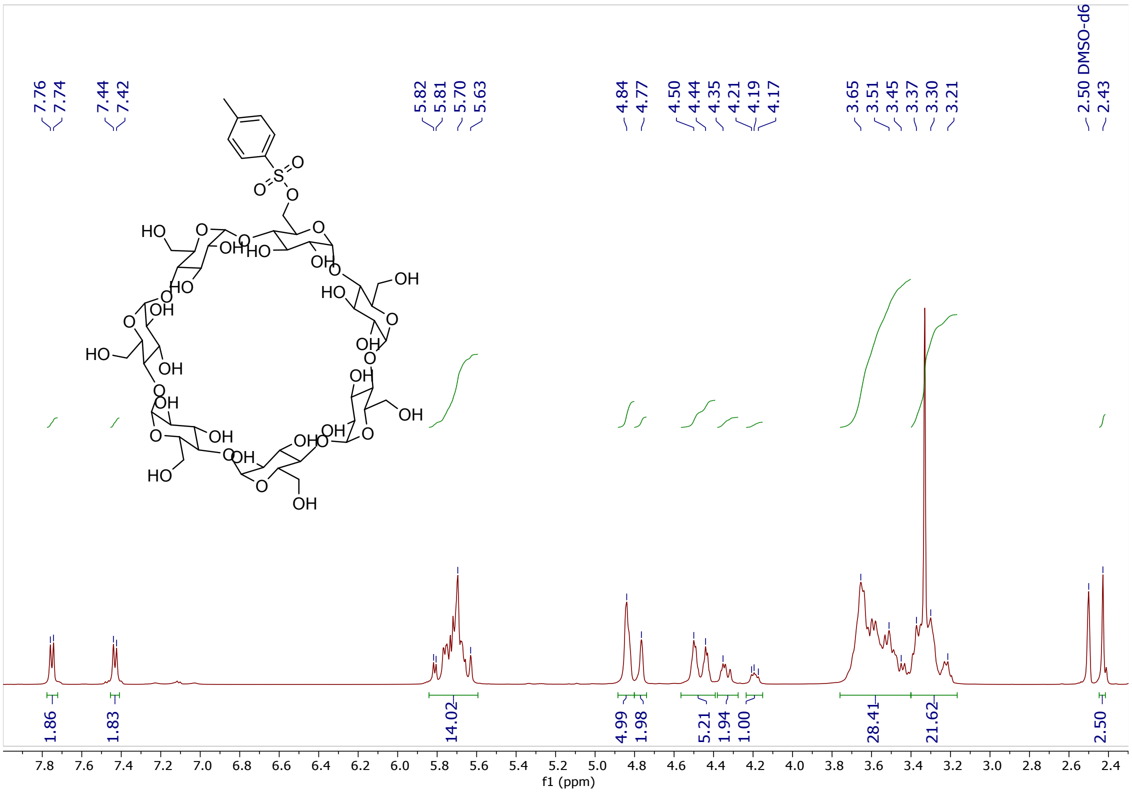

Figure S1 - ^{1}H NMR spectrum (500 MHz) of **Ts-*β*-CD** in DMSO-d_6.

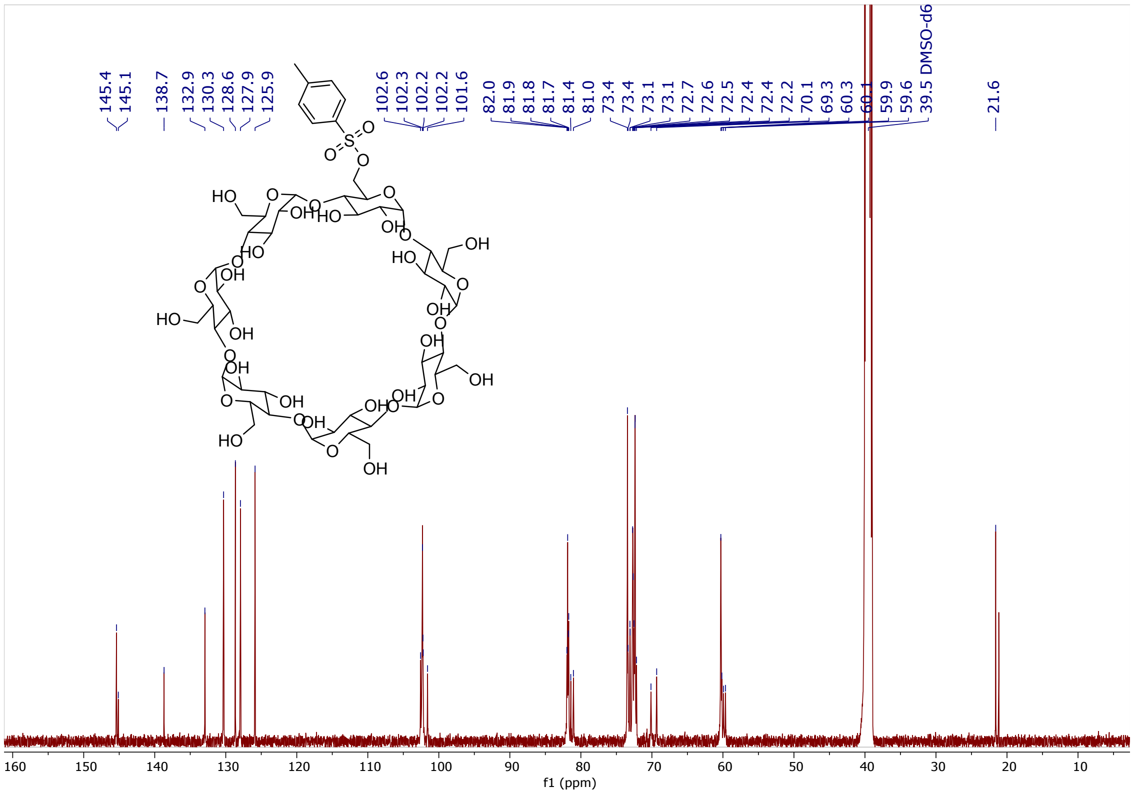

Figure S2 - $^{13}C\{^1H\}$ NMR spectrum (126 MHz) of compound **Ts-*β*-CD** in DMSO-d_6.

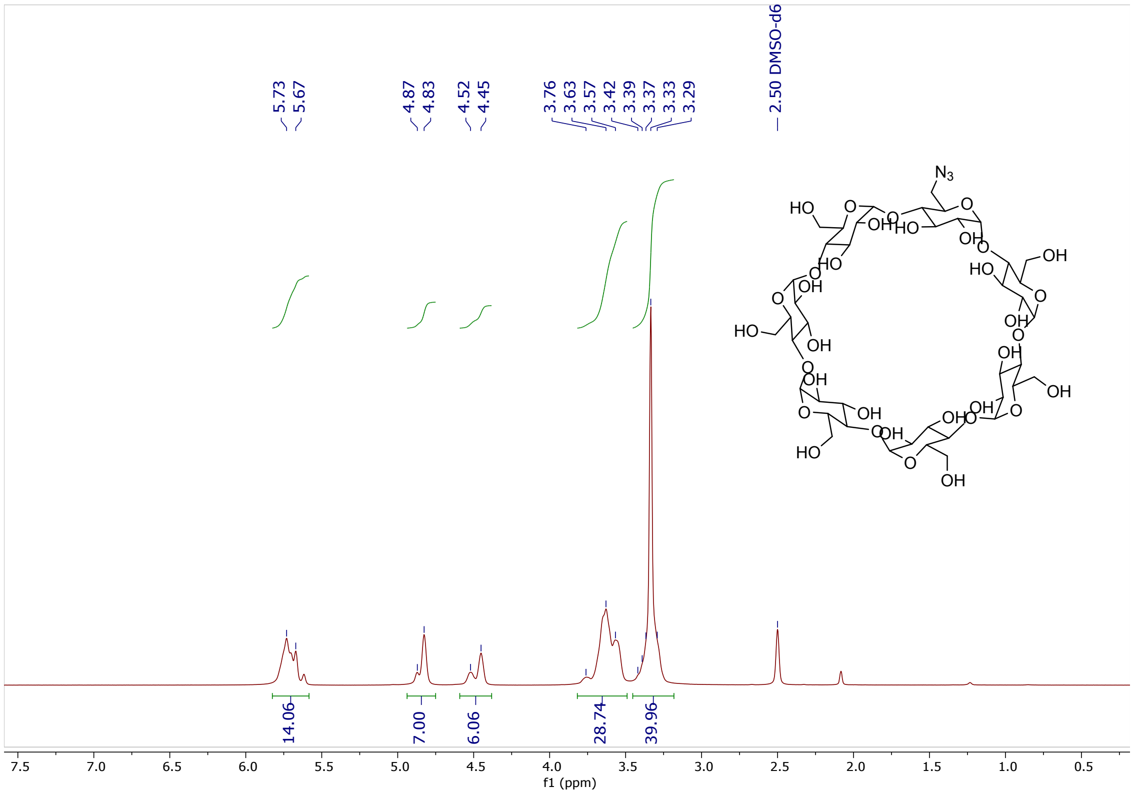

Figure S3 - ^1H NMR spectrum (500 MHz) of **N_3-*β*-CD** in DMSO-d_6.

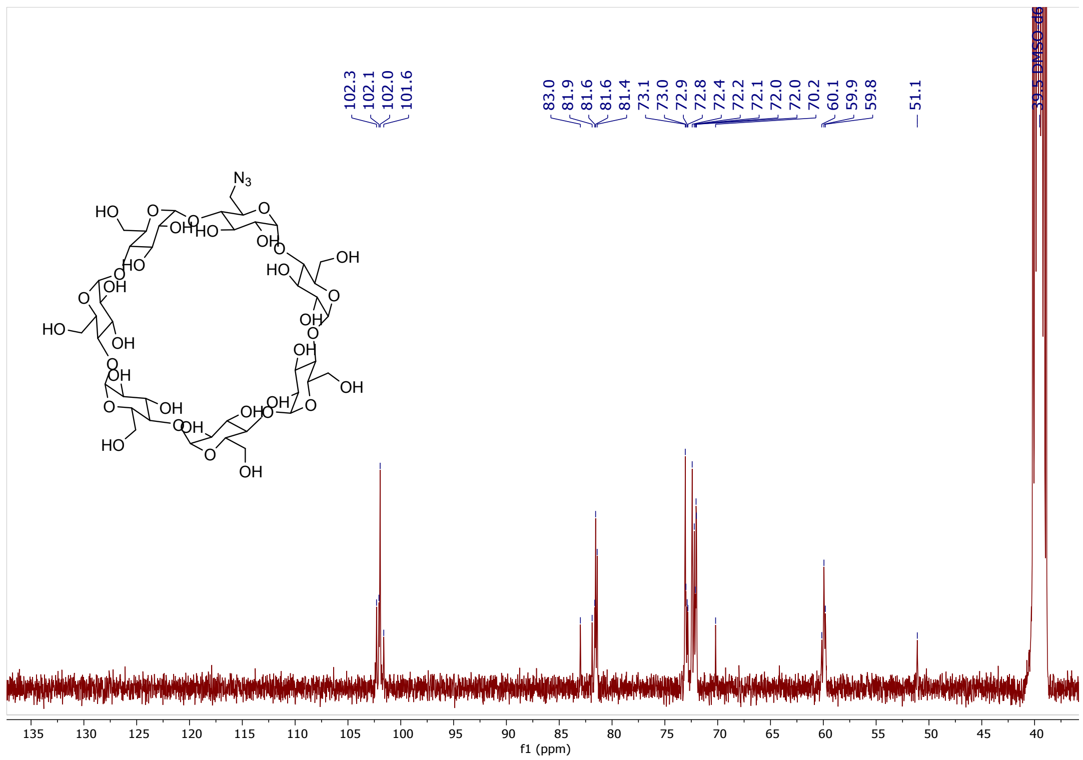

Figure S4 - $^{13}C\{^{1}H\}$ NMR spectrum (126 MHz) of compound **N_3-*β*-CD** in DMSO-d_6.

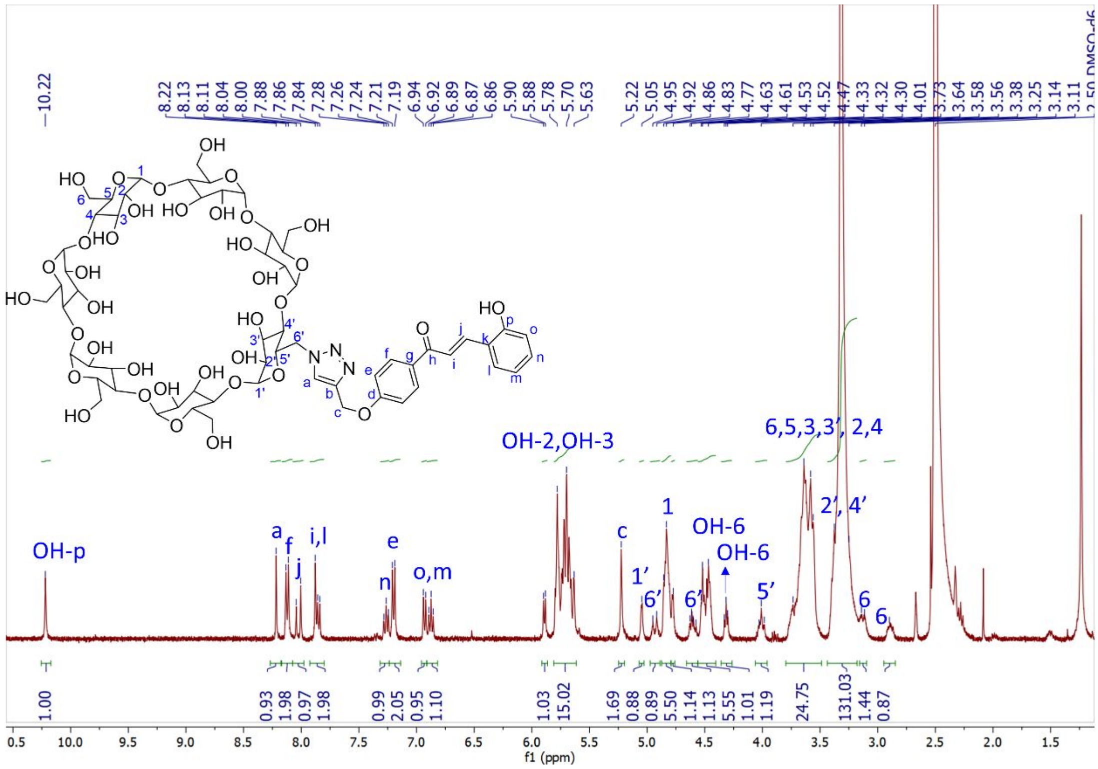

Figure S5 - ^{1}H NMR spectrum (500 MHz) of **1-Ct** in DMSO-d_6. The partial assignment of the ^{1}H signals was based on the 2D experiments presented below. Please note that we were only able to differentiate the ^{1}H corresponding to the glucose unit attached to the chalcone.

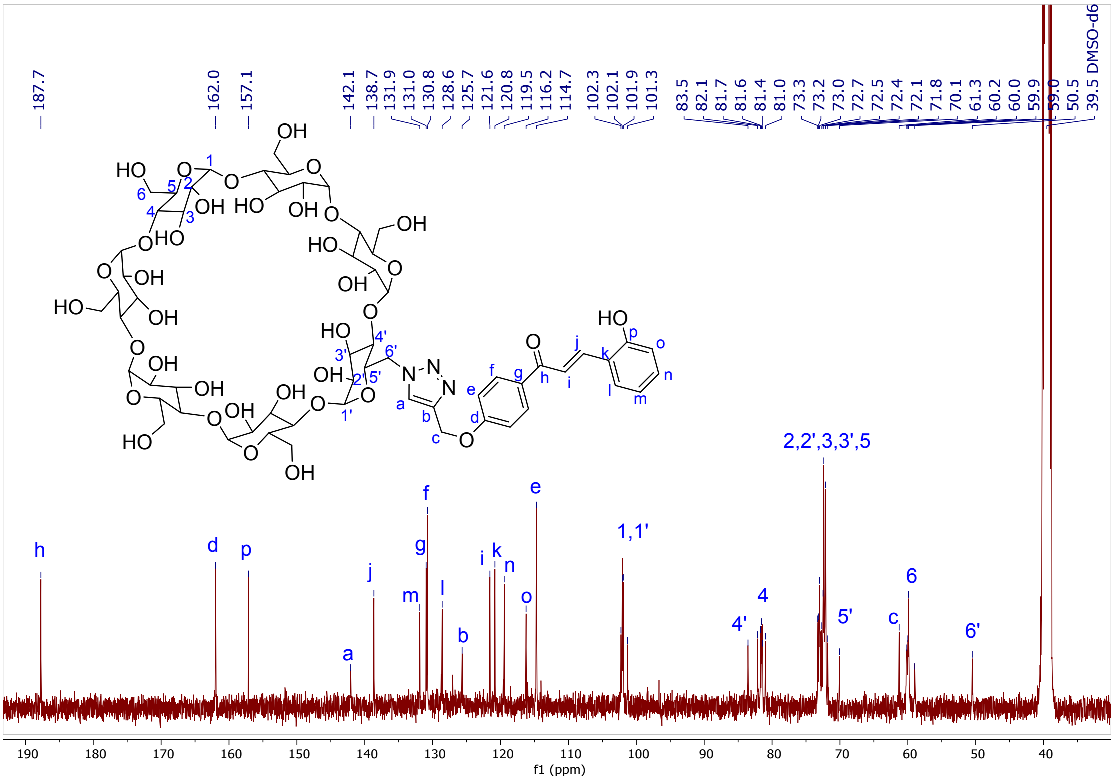

Figure S6 - $^{13}C\{^{1}H\}$ NMR spectrum (126 MHz) of compound **1-Ct** in DMSO-d_6.

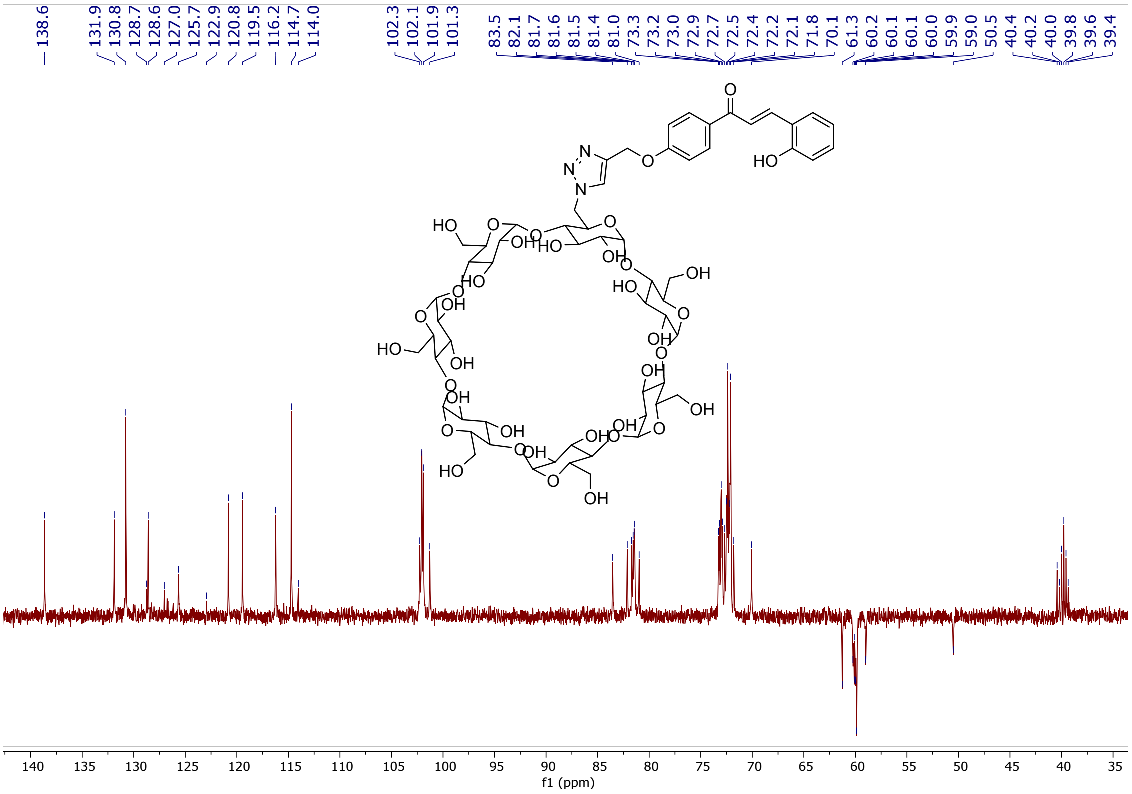

Figure S7 - DEPT135 NMR spectrum (101 MHz) of compound **1-Ct** in DMSO-d_6.

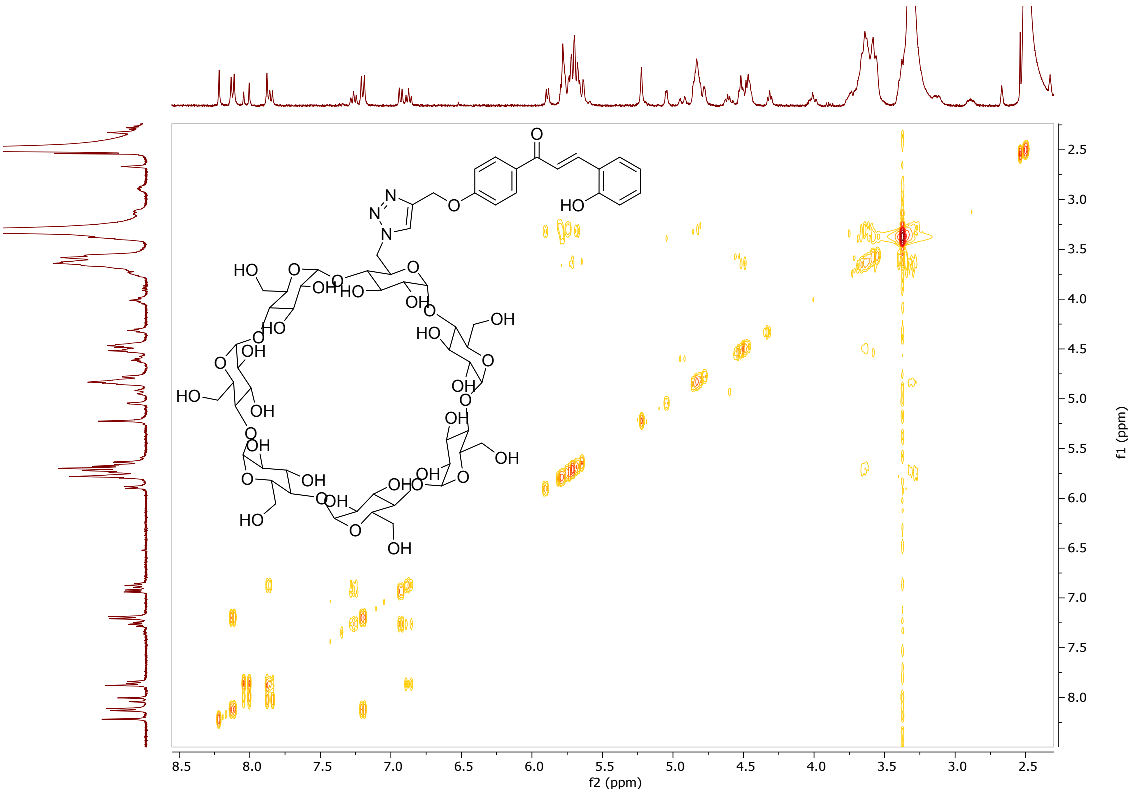

Figure S8 - COSY spectrum (400 MHz) of compound **1-Ct** in DMSO-d_6.

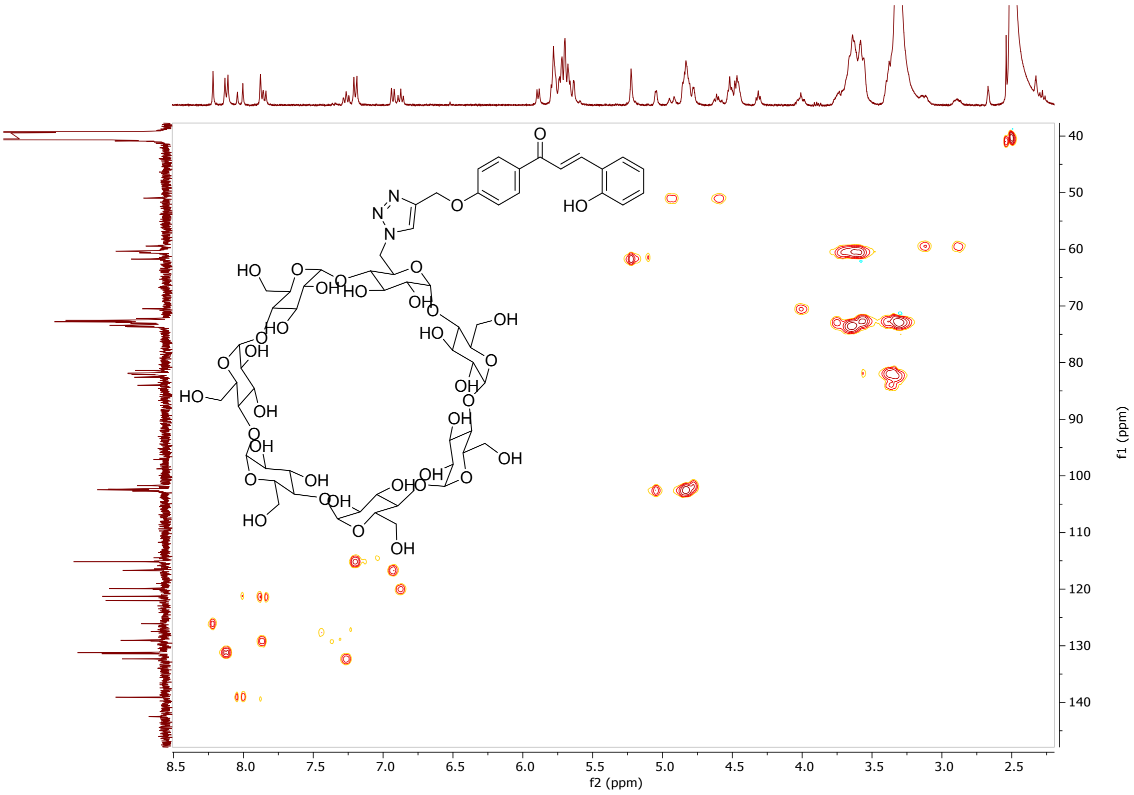

Figure S9 - HSQC spectrum (400 MHz) of compound **1-Ct** in DMSO-d_6.

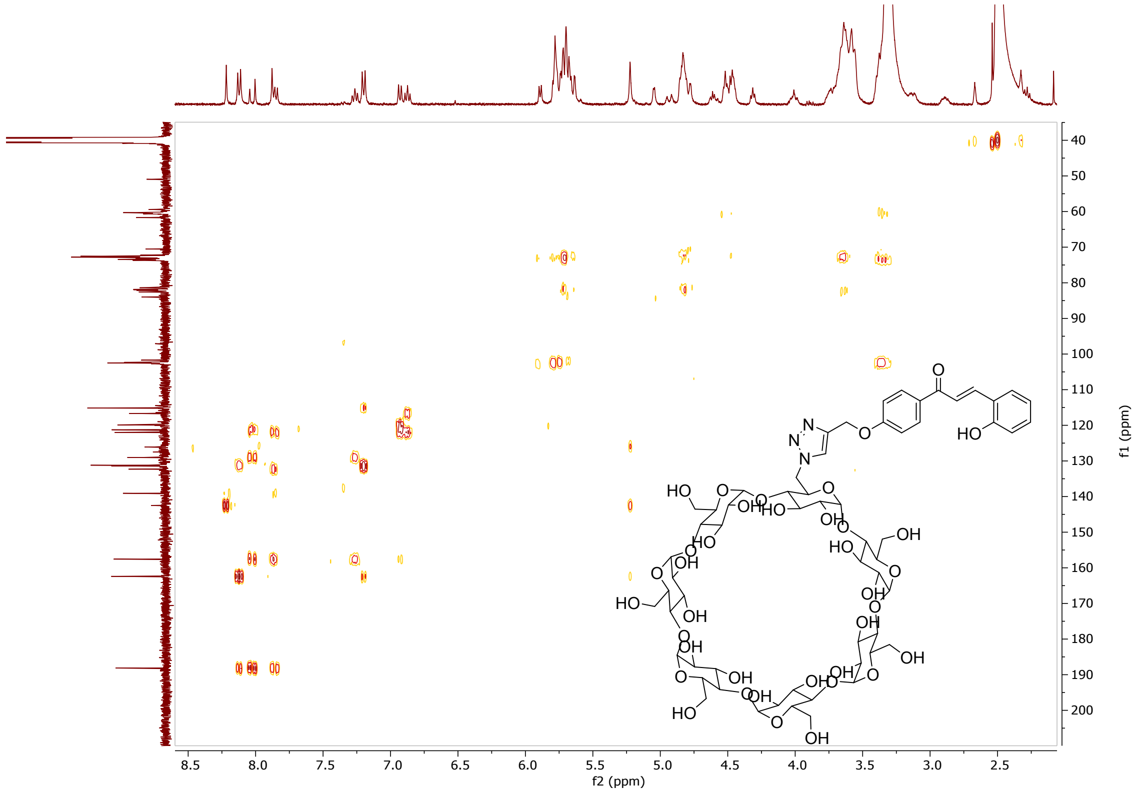

Figure S10 - HMBC spectrum (400 MHz) of compound **1-Ct** in DMSO-d_6.

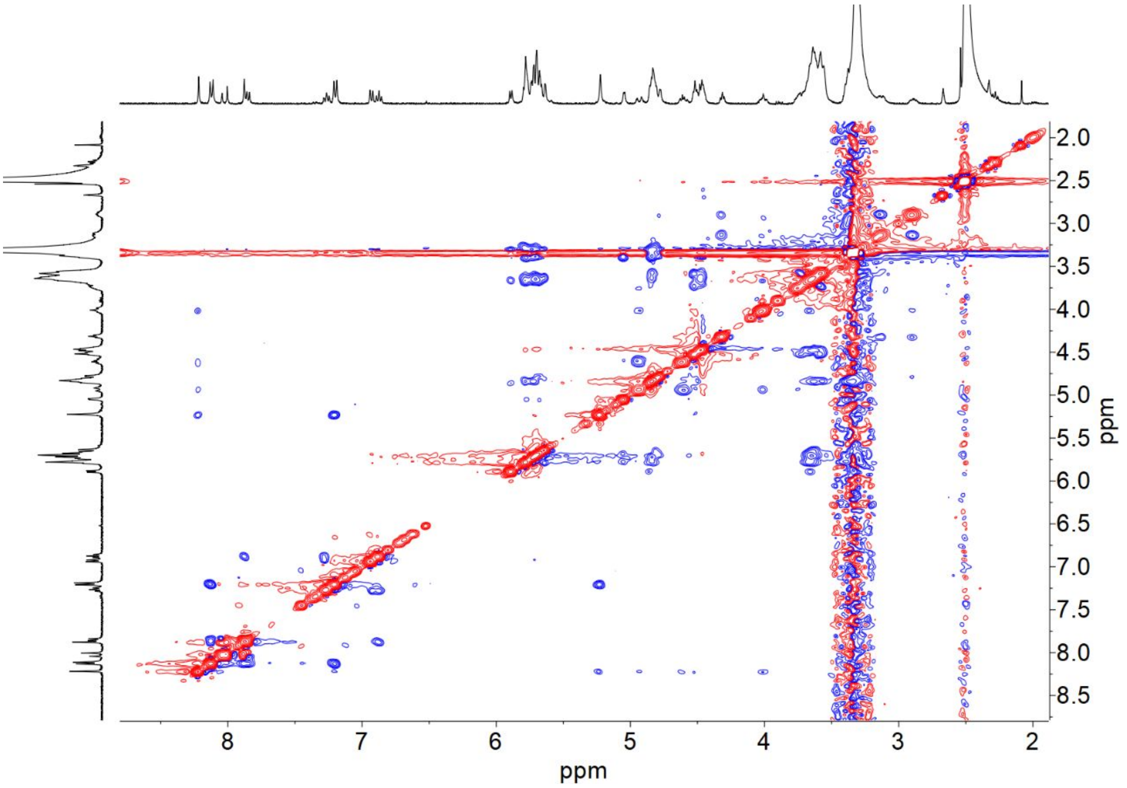

Figure S11 - ROESY (mixing time = 300 ms) spectrum (400 MHz) of compound **1-Ct** in DMSO-d_6.

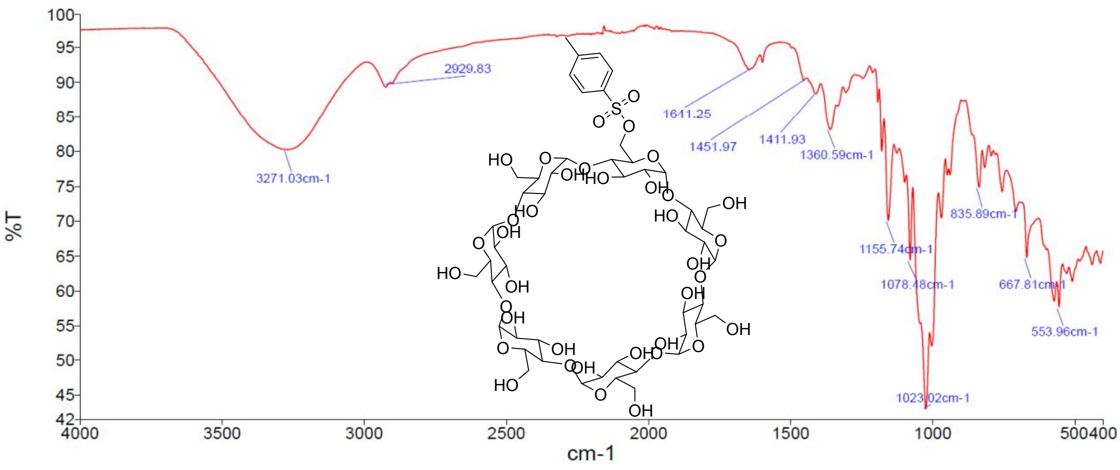

Figure S12 – FTIR spectrum of compound **Ts-*β*-CD**.

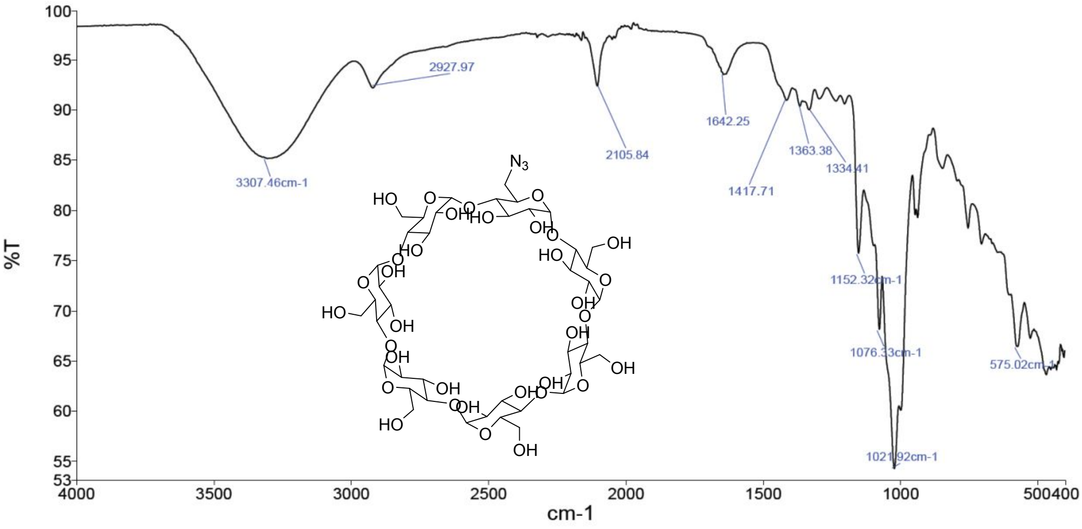

Figure S13 – FTIR spectrum of compound **N_3- *β*-CD**.

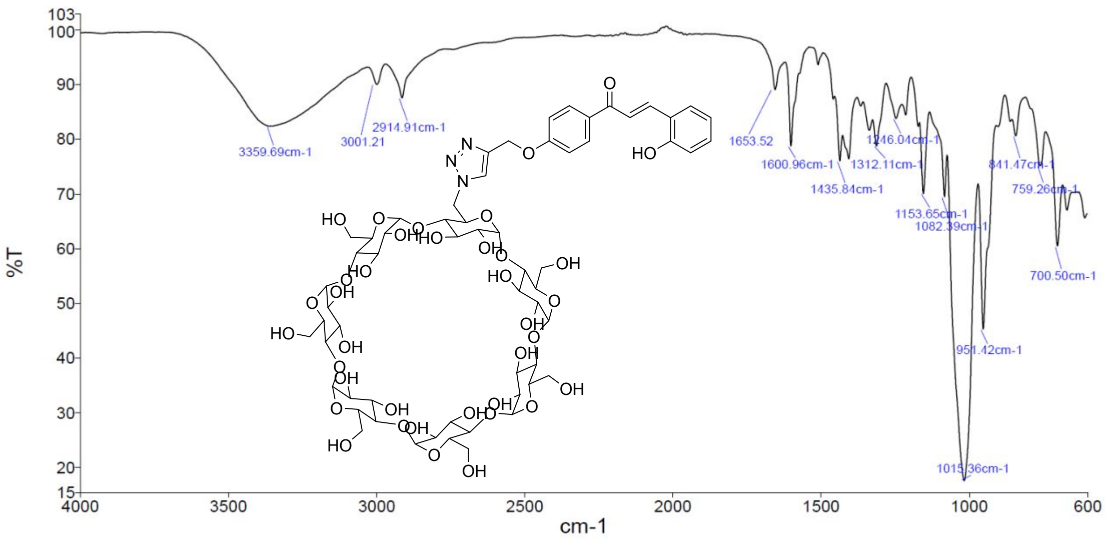

Figure S14 – FTIR spectrum of compound **1-Ct**.

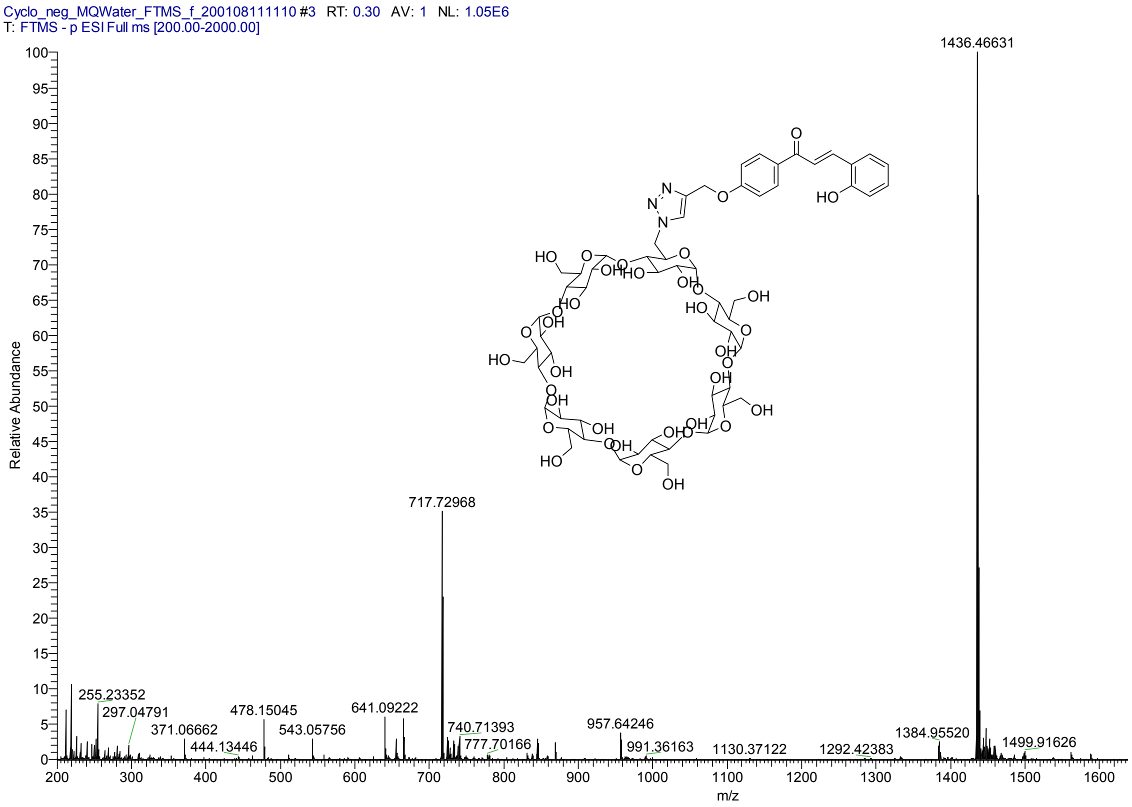

Figure S15 – HRMS spectrum of compound **1-Ct**.

2. Supplementary spectroscopic studies

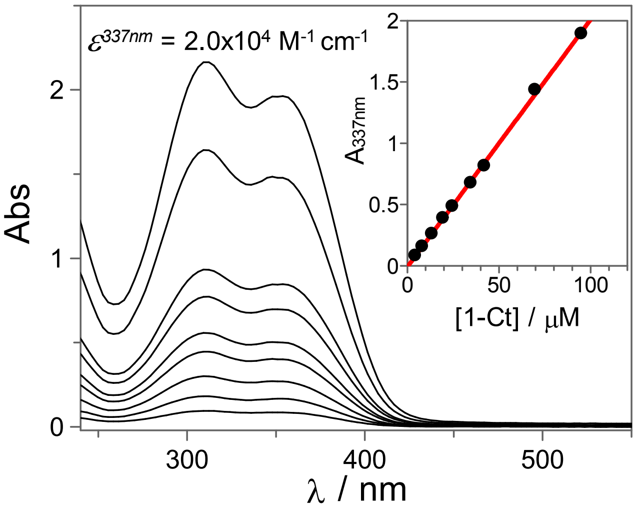

Figure S16 – Concentration-dependent UV-Vis absorption spectra of **1-Ct** (2% of DMSO in H_2O v:v) at pH = 6. The linearity observed in the absorption vs concentration plot (inset) support the formation of intramolecular self-inclusion complex (i. e. the formation of intermolecular complexes is concentration dependent resulting in deviations from linearity).

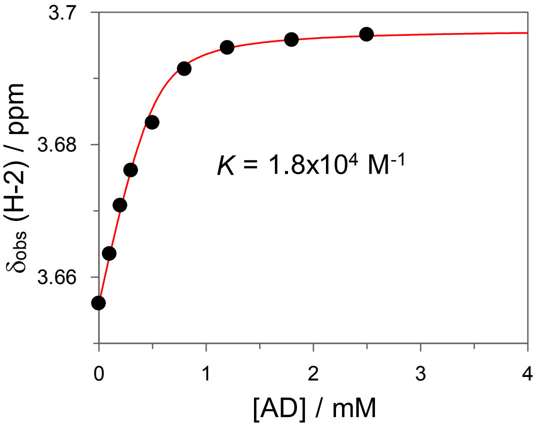

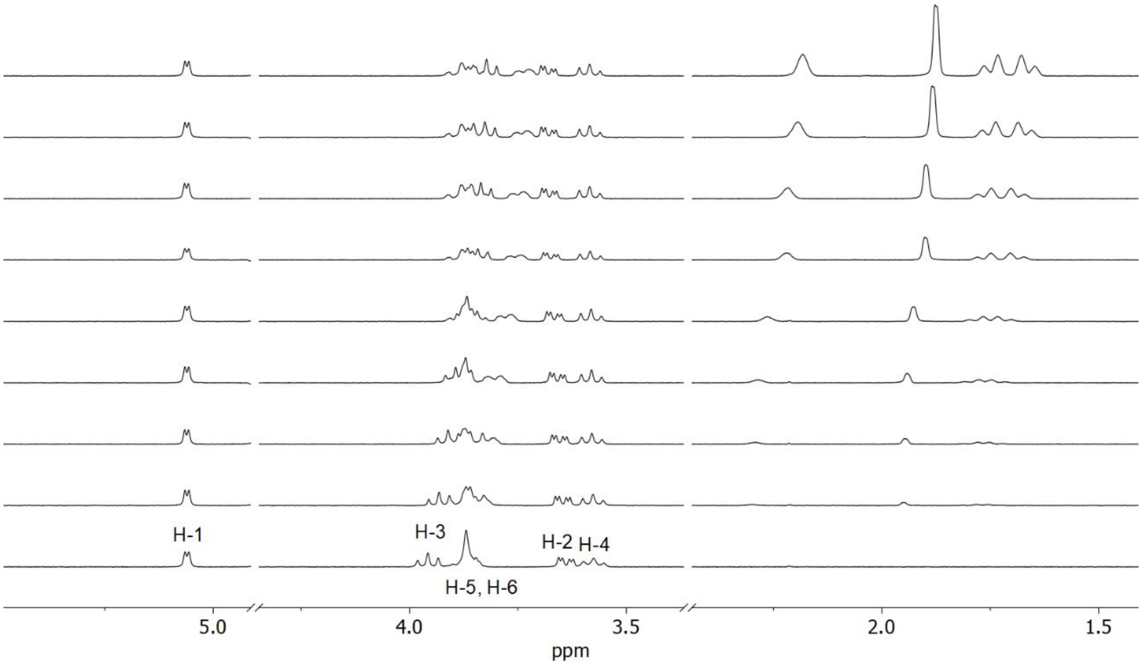

Figure S17 – ^{1}H NMR (400 MHz) host-guest titration of β-CD (0.5 mM) with increasing concentrations of adamantylammonium in D_2O.

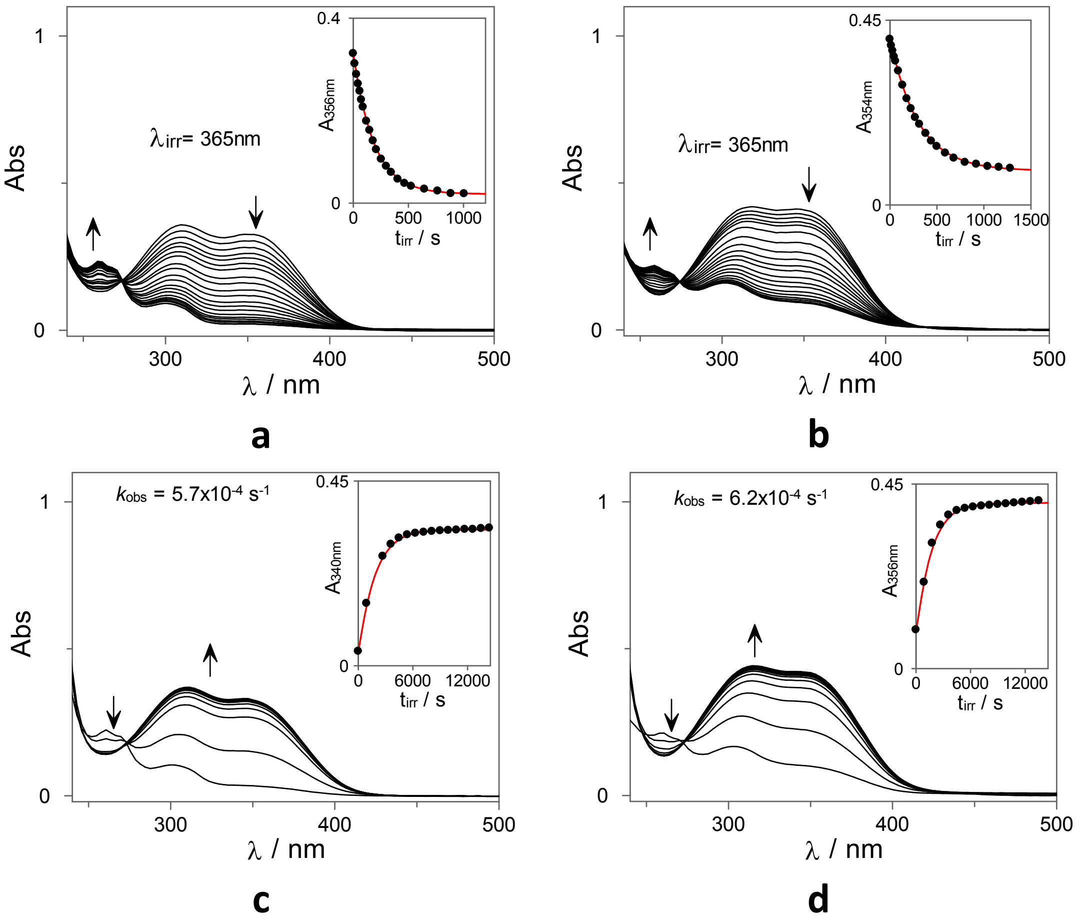

Figure S18 – (**a**) Spectral variations observed upon UV-irradiation (365 nm) of compound **1-Ct** (28 µM 2% of DMSO in H_2O v:v) at pH = 5.5. (**b**) the same in the presence of 10 mM of AD at pH = 5.6. (**c**) Thermal recovery, at 60 °C, of the *trans*-chalcone from the photogenerated hemiketal and *cis*-chalcone forms in the absence and (**d**) in the presence of 10 mM of AD.

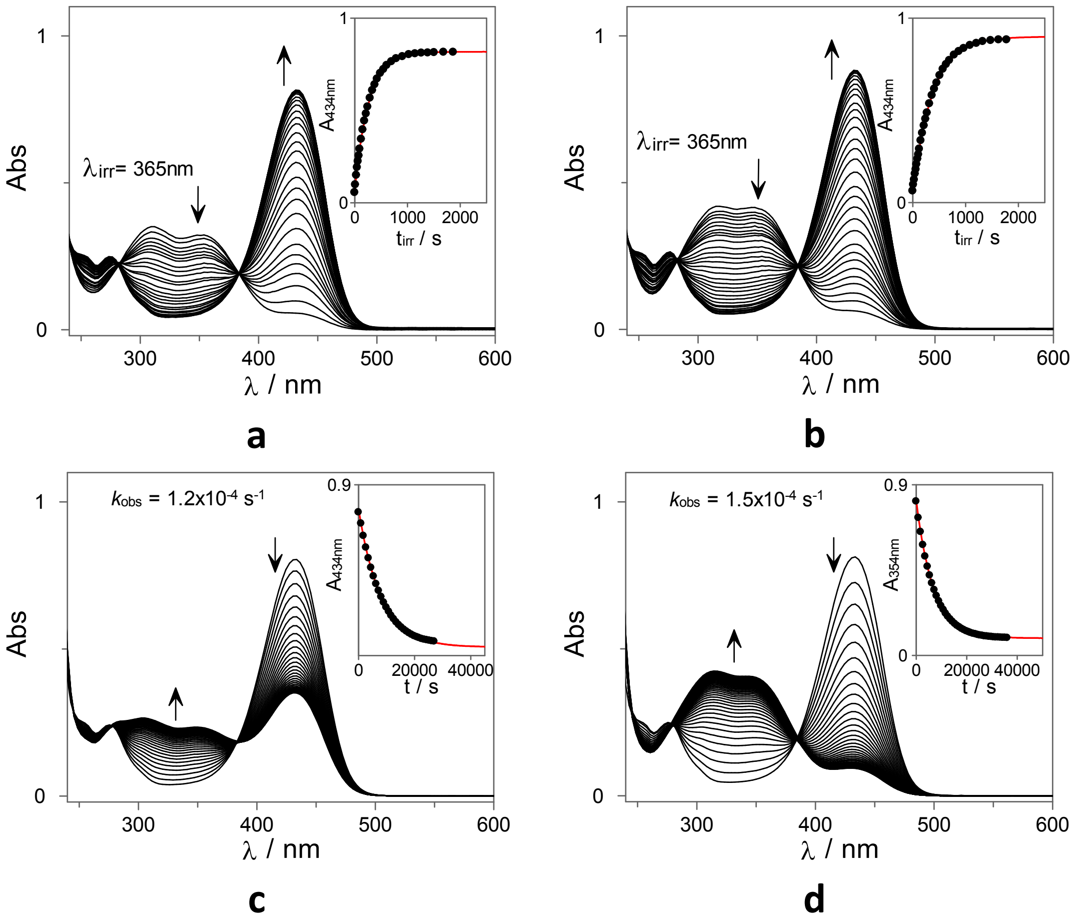

Figure S19 – (**a**) Spectral variations observed upon UV-irradiation (365 nm) of compound **1-Ct** (28 μM 2% of DMSO in H_2O v:v) at pH = 1.1. (**b**) the same in the presence of 10 mM of AD at pH =2.3. (**c**) Thermal recovery of the photogenerated flavylium form of **1** (28 μM 2% of DMSO in H_2O v:v) at 60 °C and pH = 1.1. (**d**) the same in the presence of 10 mM of AD at pH = 2.3.

Inspection of the photostationary state composition by reverse pH jumps

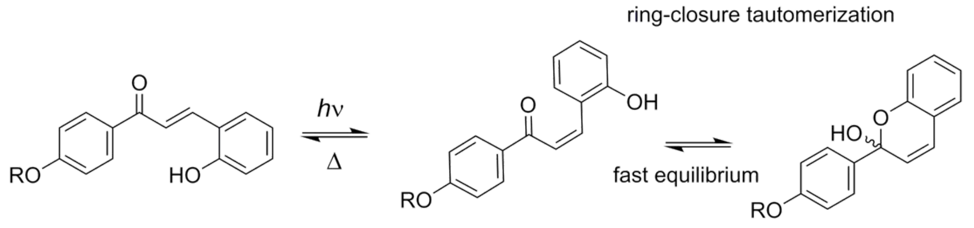

PSS composition:	2%	7%	91%
PSS composition with AD:	13%	19%	68%

Scheme S1 – *trans*-Chalcone photochemistry at slightly acidic/neutral pH conditions.

Upon acidification, the mole fractions of hemiketal and *cis*-chalcone are immediately (sub seconds time-scale) converted into the flavylium cation (which absorbs at longer wavelengths) while the *trans*-chalcone is converted in a much longer time scale through a thermal reaction channel or, as in the present case, through further irradiation in acidic conditions (i.e. at pH values where the flavylium cation formation is quantitative). As can be observed from Figure S20, acidification of an irradiated solution of **1-Ct** leads to the appearance of an absorption band with a maximum at 434 nm that is ascribed to the flavylium cation (**1-AH$^+$**). Upon further irradiation at 365 nm in acidic conditions, this band increases by 2% which is assigned to the *trans*-chalcone at the PSS. Thus, 98% corresponds to an equilibrium mixture of *cis*-chalcone and hemiketal. On the other hand, similar experiments in the presence of AD shows a larger fraction (13%) of *trans*-chalcone at the PSS. This points out to the preferential stabilization of the hemiketal form inside the cyclodextrin cavity driving the *cis*-chalcone/hemiketal equilibrium towards this last species.

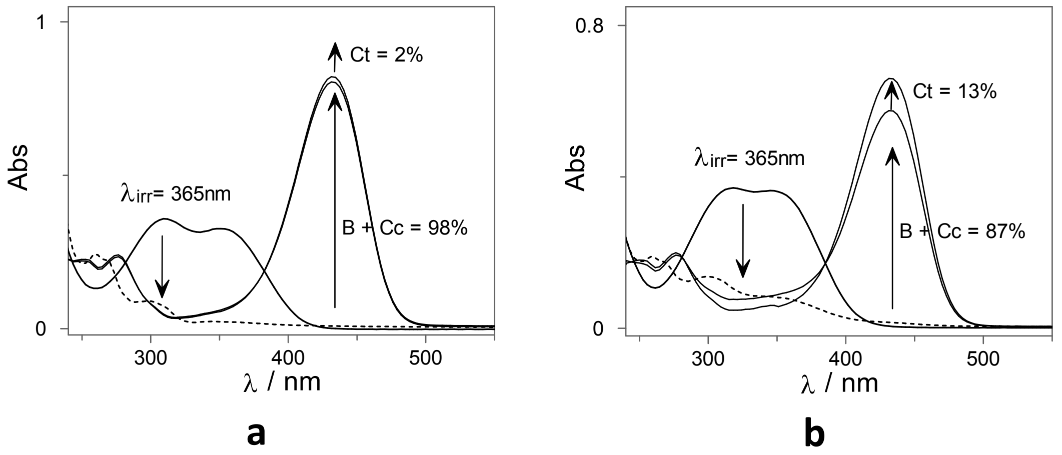

Figure S20 – (a) The irradiation-pH jump-irradiation experiment employed to estimate the fraction of *trans*-chalcone at the photostationary state. **1-Ct** (28 μM 2% of DMSO in H_2O v:v) pH = 5.6. (b) the same in the presence of 10 mM of AD at pH = 5.5. The dotted lines correspond to the PSS spectra.

The composition of the PSS can be further examined through the monitorization of the reverse pH-jumps by stopped-flow. The method takes advantage of the fact that at very acidic conditions the hemiketal is rapidly converted into the flavylium cation while the *cis*-chalcone converts slower (via hemiketal) giving rise to biexponential kinetics with the pre-exponential factors corresponding to the mole fraction of these species (since the reaction is monitored in the visible region of the spectrum where only the flavylium cation absorbs).[1,2] As can be observed from Figure S21 the PSS corresponds mainly to the hemiketal (B= 93%) containing a smaller fraction of *cis*-chalcone (Cc = 7%). In the presence of 10 mM of AD, the fraction of these last species increases (Cc = 22%) at the expenses of the former (B = 78%). This trend increases for higher concentrations of AD as shown in Figure S22.

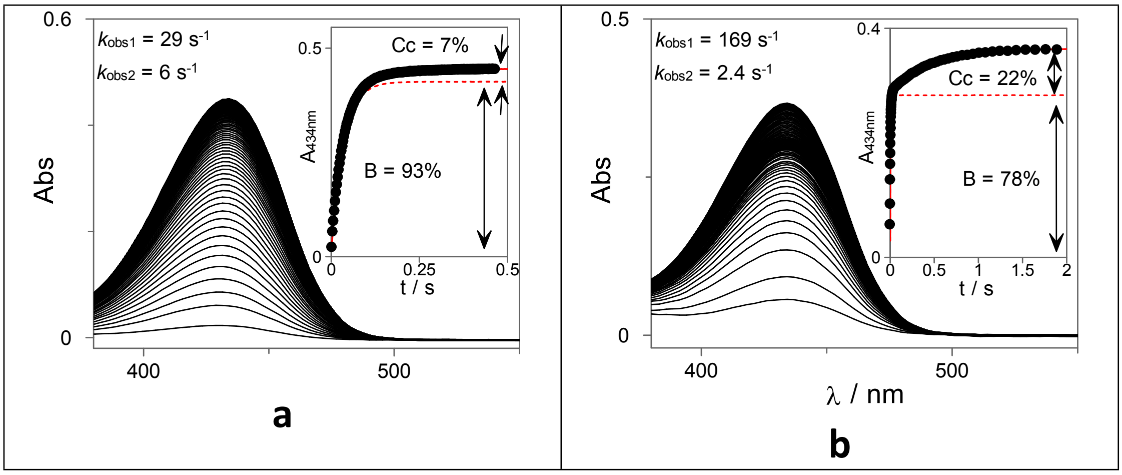

Figure S21– (a) Reverse pH jump from a solution of **1** (14 μM 2% of DMSO in H_2O v:v) at pH = 5.6 in photosationary state to pH = 0.6. (b) the same in the presence of 10 mM of AD from pH = 5.5 to pH = 1.

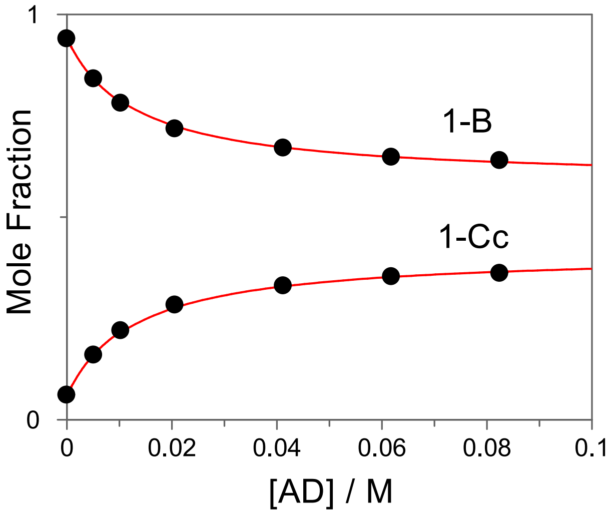

Figure S22 – Mole fraction of **1-B** and **1-Cc**, determined from reverse pH jump experiments monitored by stopped-flow, in presence of increasing amounts of AD.

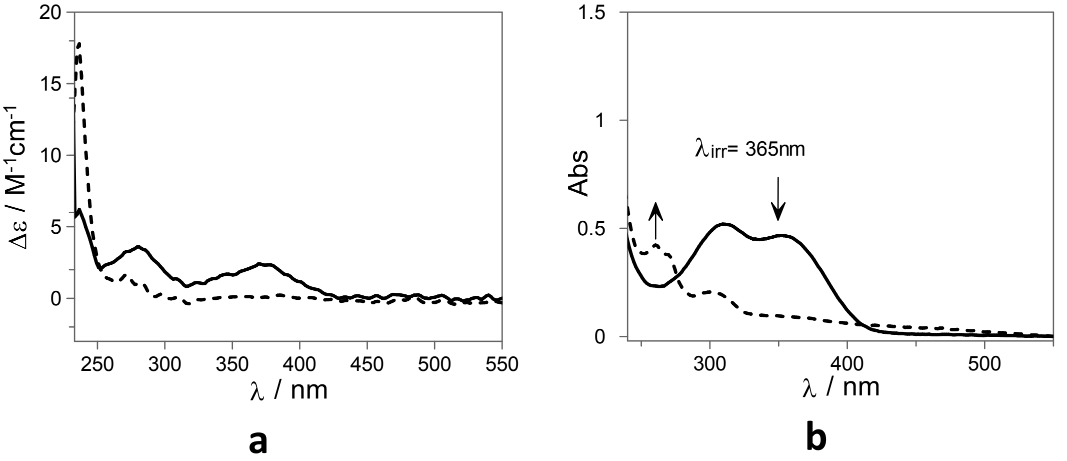

Figure S23 – (**a**) Electronic circular dichroism and (**b**) absorption spectra of **1** (28 μM 2% of DMSO in H_2O v:v) at pH = 5.6 before (full line) and after irradiation (dotted line).

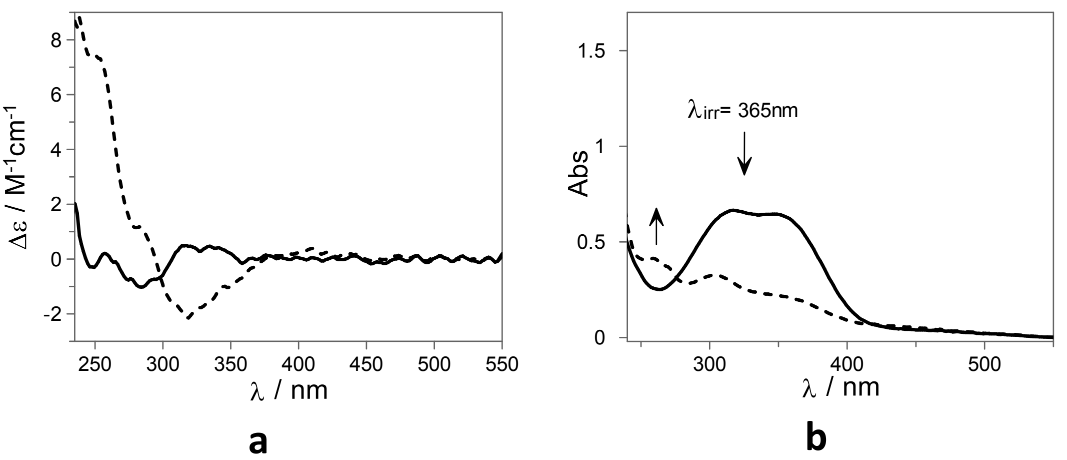

Figure S24 – (**a**) Electronic circular dichroism and (**b**) absorption spectra spectra of **1** (28 μM 2% of DMSO in H_2O v:v) in the presence of 10 mM of AD at pH = 5.6 before (full line) and after irradiation (dotted line).

3. Computational studies

DFT conformational studies of the chalcone/flavylium.

The stability of different structural possibilities for the chalcone arm was explored by DFT calculations performed at the M062X/6-311+G* level using Gaussian09. For this purpose, gas phase geometrical optimizations followed by calculation of vibrational frequencies for characterizing the structures as energy minima has been performed. The results obtained indicate, as expected, a higher preference for the *trans* conformation over the *cis* one (4.7 kcal/mol) (Table S1). On the other hand, two different non-planar geometrical structures for the *trans* chalcone characterized by a *syn* or *trans* O=C···C—O periplanar disposition were found as stable energy minima and the energy difference between both dispositions is almost negligible (0.63 kcal/mol). For all these conformations, the triazole unit is coplanar with the intermediate carbon atom ring (R1). The planar moiety formed by the triazole unit and the adjacent R1 ring forms an angle between 20-60º with the plane defined by the phenyl ring at the opposed edge of the molecule (R2).

Table S1. Conformational study of chalcone. Relative energies.

Conformer	Total energy (a.u.)	Relative energy (kcal/mol)	O=C⋯C–O torsion	Angle between rings R1/R2
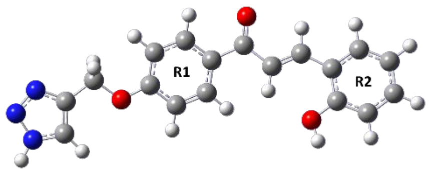 trans (1)	-<br>1084.644468	**0.00**	-171.5º	21.3º
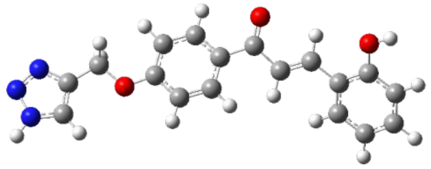 trans (2)	-<br>1084.645469	**0.63**	30.9º	46.6º
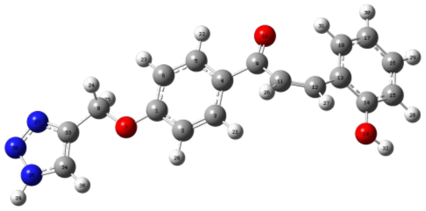 cis	-<br>1084.638021	**4.67**	175.4º	57.1º

For the flavylium cation, there are two different relative dispositions between the triazole unit and the two fused rings that can arise from the photochemical reaction of the chalcone and both were characterized as energy minima (Table S2). These structures have virtually the same relative stability and present a planar region containing the three aromatic carbon rings with the triazole unit almost perpendicular to the planar ring moiety. Although both forms can be interchangeable by rotation through the central C-C bond connecting the aromatic systems or the O-C bond adjacent to the triazole unit, the energy barriers predicted from restrained geometry energy calculations for both rotations are rather high (around 10 kcal/mol).

Table S2. Conformational study of the flavylium cation. Relative energies.

Conformer	Total energy (a.u.)	Relative energy (kcal/mol)	Angle between the triazole and aromatic unit planes
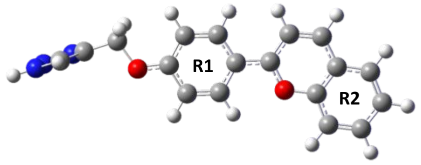	-1008.602479	**0.00**	87.5º
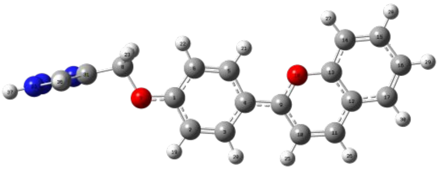	-1008.602469	**0.01**	87.1º

Molecular Dynamics

Molecular dynamics (MD) simulations were performed using GROMACS 2020.3. Initial structures for systems containing the trans-chalcone arm inside and outside the cyclodextrin cavity were built employing the coordinates in 3M3R (PDB code) for the cyclodextrin and the previously DFT optimized structures for the *trans*-chalcone. The CHARMM36 force field was employed to describe the cyclodextrin moiety and initial parameters for the trans-chalcone were obtained with the CGenFF program. Further refinement of the proposed parameters with larger penalties was carried out using the ffTK tool. The cyclodextrin-chalcone system was solvated with water modelled with the TIP3P model in cubic boxes with a side-length of 35 Å. These structures were energy minimized in vacuum with the positions of the heavy atoms restrained with 1000 kJ mol^{-1} nm^{-2}, followed by NPT and NVT equilibration simulation of 500 ps with restraints on the heavy atoms of cyclodextrin (1000 kJ mol^{-1} nm^{-2}). Production run for 50 ns using a 2 fs time step and saving configurations each 10 ps. The temperature was kept constant at 300 K with the Nosé−Hoover thermostat using a coupling constant of

1 ps and the pressure was set to 1 bar with the Parrinello−Rahman barostat by isotropic coupling using a coupling constant of 5 ps. The same procedure was followed in additional MD simulations for the cyclodextrin comprising *cis*-chalcone, hemiketal, or flavylium arm.

According to the MD simulations, the chalcone branch shows a great conformational variability moving up and down inside the *β*-CD without leaving the cavity. Thus, the results in Figure S25 indicate that the distance between the *β*-CD center of mass (com) and the center of the phenyl ring R2 oscillates approximately between 2 and 7 Å. The dominant conformations correspond to those with larger distances, meaning that the chalcone branch is deeply buried in the *β*-CD with the R2 ring outside the cavity and the R2 ring plane forming an angle around 50º with a vertical axis passing through the *β*-CD center. In these conformations, the R1 ring is clearly inside the cyclodextrin cavity in a disposition almost parallel to the central axis (see Figure 2 in the manuscript).

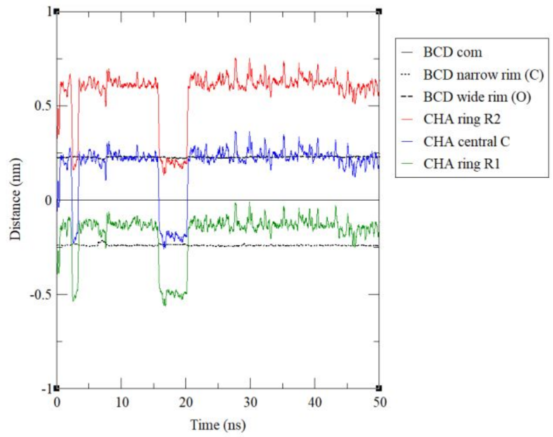

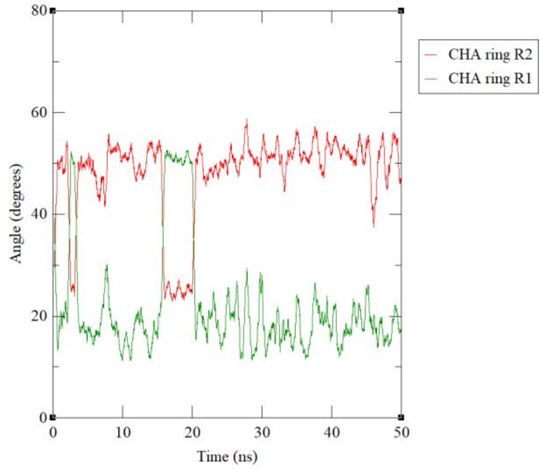

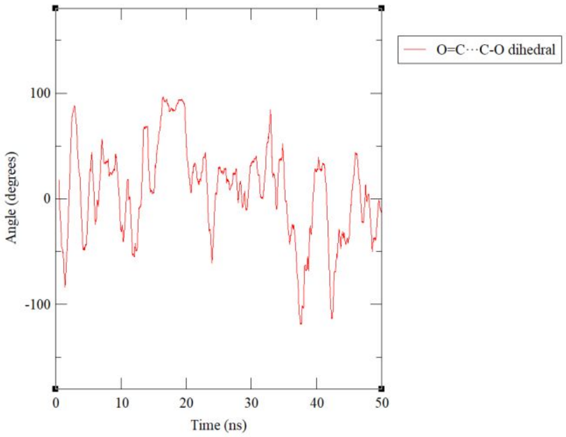

Figure S25 – (Up Left) Selected distances for the chalcone arm included in β-CD taking the β-CD center of mass (com) as origin for measuring distances: the dashed black bottom line is the distance to the β-CD narrow rim formed by the carbon atoms of the hydroxy groups; the black dotted upper line is the distance to the β-CD wide rim formed by the oxygen atoms of the hydroxy groups; the red line is the distance to the com of

the chalcone ring R2; the green line is the distance to the com of the chalcone ring R1; the blue line is the distance to the central carbon atom of the chalcone arm. (Up Right) Selected angles for the chalcone rings with the central axis of the *β*-CD. (Bottom) O=C···C—O dihedral angle.

These buried conformations have larger occurrences than those with shorter distances between the com of the *β*-CD and the center of the R2 ring, which correspond to structures where only half of the chalcone arm is included in the *β*-CD cavity and, consequently, the R2 ring is inside the cyclodextrin in a disposition almost parallel to the vertical axis of *β*-CD (see Figure 2 in the manuscript). Interestingly, the vertical position of the chalcone arm seems to be correlated with the relative disposition between the carbonyl and hydroxy groups. Thus, when the phenyl ring R2 is clearly inside the *β*-CD, the C=O and OH groups tend to be in a skew orientation with angles around +100º, which suggests that there is not space enough for the chalcone arm to adopt either a *syn*- or *anti*-periplanar disposition. However, when R2 is clearly above the large rim of the β-CD, there is a large conformational flexibility around the planar dispositions with the C=O and OH groups in syn. In these conformations, the R1 ring tends to be parallel to the central axis, while the R2 ring bends and presents angles around 50º.

Since experimental data suggest that the chalcone arm is displaced from the *β*-CD cavity upon addition of AD, we also performed MD simulations with the chalcone branch outside the cavity starting from different initial dispositions. As expected, our results show that the chalcone arm presents a much larger conformational variability outside the *β*-CD cavity. However, regardless of the starting configuration, there is a significant occurrence of dispositions where the chalcone arranges approximately almost parallel to the *β*-CD outer wall (Figure S26).

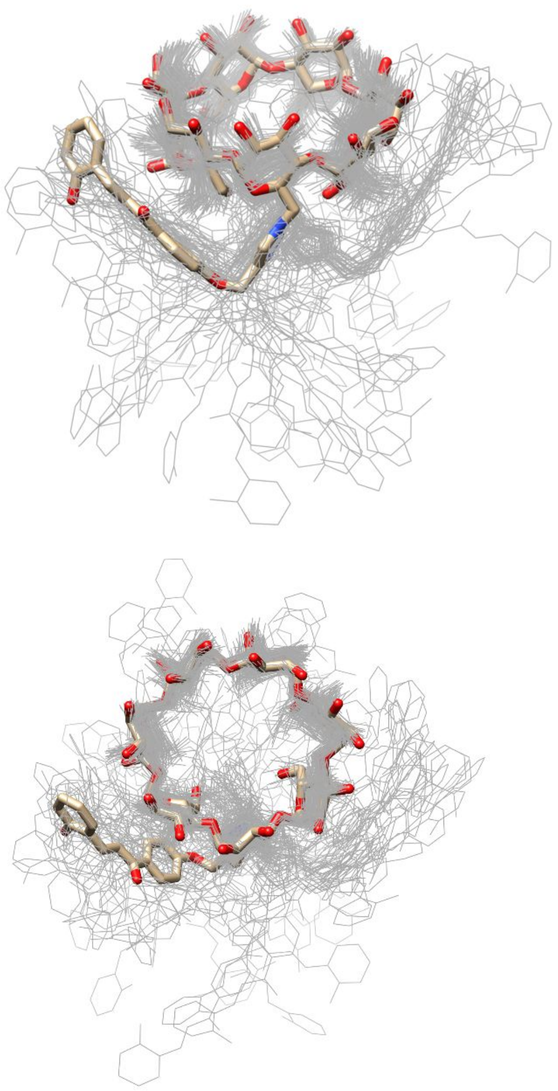

Figure S26 – Conformations of the chalcone arm outside the *β*-CD cavity.

We have also carried out MD simulations with the chalcone arm inside the cavity increasing the temperature from 300 to 500 K. The distance from the *β*-CD com to the R2 ring shown in Figure S27 shows that, as expected, conformational flexibility increases from 300K to 450K since the occurrence of conformations where the chalcone is deeply buried within the cavity increases. Interestingly, around 480K-490K, there is a huge increase in the distance between the *β*-CD com and the R2 ring which indicates that the chalcone leaves the *β*-CD.

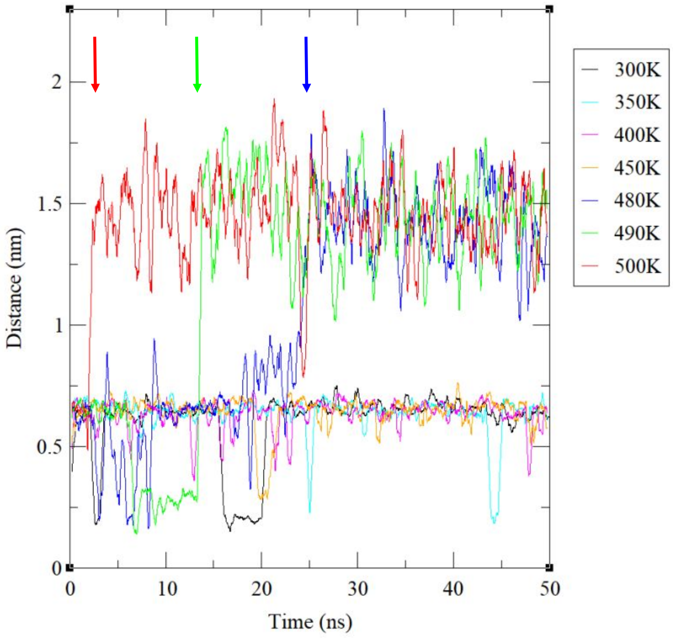

Figure S27 – Selected distances for the chalcone R2 to the β-CD center of mass at different temperatures. The arrow lines indicate that the chalcone arm leaves the cyclodextrin cavity.

MD simulations were also performed for systems including the flavylium motif inside and outside the β-CD cavity. It must be noted here that we performed separated MD simulations taking as initial dispositions for the flavilium branch the two different arrangements of the fused rings (see table S2) that can arise from the photochemical reaction of the chalcone. We observed that the rotation of the flavylium arm is somewhat hindered within the β-CD cavity as suggested by the small variations found for the dihedral angles formed between the central aromatic rings and between the triazole unit and its adjacent aromatic ring (see Fig S28). As a consequence, the initial configuration of the flavylium arm is maintained during our 50 ns simulations. On the other hand, the up and down mobility of the flavylium cation inside the cavity is smaller than for the

chalcone arm as can be concluded from the smaller variations obtained for the selected distances shown in Figure S28. Also, as a consequence of this reduced mobility, the organic arm adopts a planar disposition tilted between 30 and 50º with respect to the vertical axis of the β-CD.

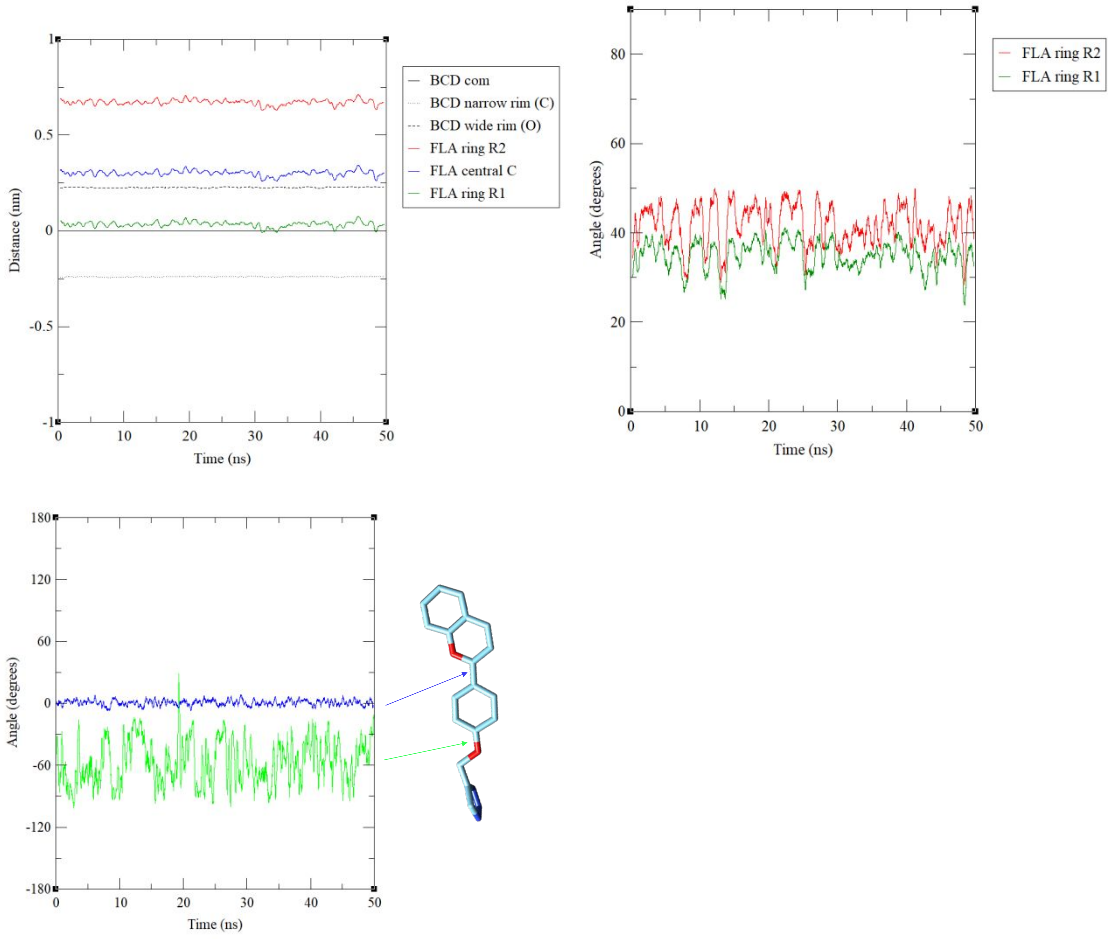

Figure S28 - (Left) Selected distances for the flavylium arm included in β-CD taking the β-CD center of mass (com) as origin for measuring distances: the dashed black bottom line is the distance to the β-CD narrow rim formed by the carbon atoms of the hydroxy groups; the black dotted upper line is the distance to the β-CD wide rim formed by the oxygen atoms of the hydroxy groups; the red line is the distance of the flavylium ring R2 to the com; the green line is the distance of the flavylium ring R1to the com; the blue line is the distance to the central carbon atom of the flavylium arm. (Right) Selected

angles for the flavylium rings with the central axis of the *β*-CD. (Bottom) Central (blue) and triazole (green) dihedral angles in the flavilium branch.

As for the chalcone derivative, MD simulations of models with different initial dispositions of the flavylium branch outside the *β*-CD cavity indicate a large flexibility for the organic arm around the *β*-CD. Here, in a similar way as for the chalcone case, it was observed that, despite the large mobility, there is a high occurrence of the flavylium arm adopting configurations approximately parallel to the *β*-CD wall (see Figure S29), regardless of the initial disposition of the flavylium fused rings.

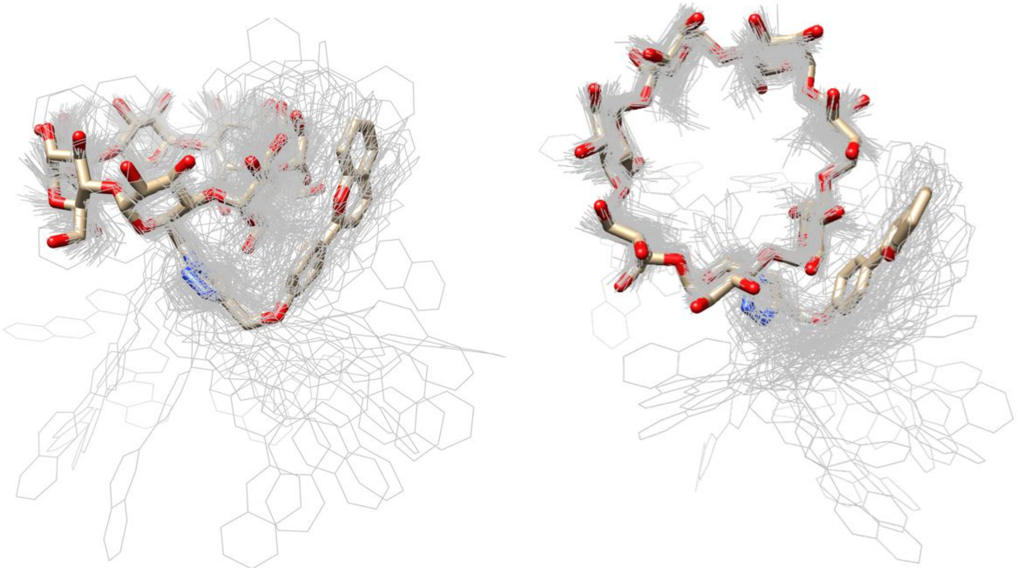

Figure S29- Conformations of the chalcone arm outside the *β*-CD cavity.

Since upon irradiation the *trans*-chalcone leads to the formation of *cis*-chalcone, we also performed MD simulations for those systems with the organic arm inside and outside the *β*-CD. The mobility of the *cis*-chalcone arm inside the cavity is quite similar to that of the trans-chalcone case: Thus, the distances in Fig. S30 reflect the up and down movement of the branch, with a high occurrence for those dispositions with larger

distances between the β-CD com and the R2 ring (around 5 A) corresponding to conformations where the cis-chalcone arm is deeply buried in the cavity and with the R2 aromatic ring clearly tilted (around 60º) from the central vertical axis of the cyclodextrin cavity. Furthermore, according to the values for the central O=C···C−O dihedral angle, the chalcone arm also rotates considerably inside the β-CD cavity. Interestingly, the values for these central O=C···C−O angles can be related to the enantiomeric forms of the hemiketal obtained from the *cis*-chalcone: Thus, conformations with positive dihedral angles will generate *R* enantiomers of the hemiketal branch after ring closure (pro-*R* conformations) while negative values of the dihedral angles will produce *S* enantiomers (pro-*S* conformations).

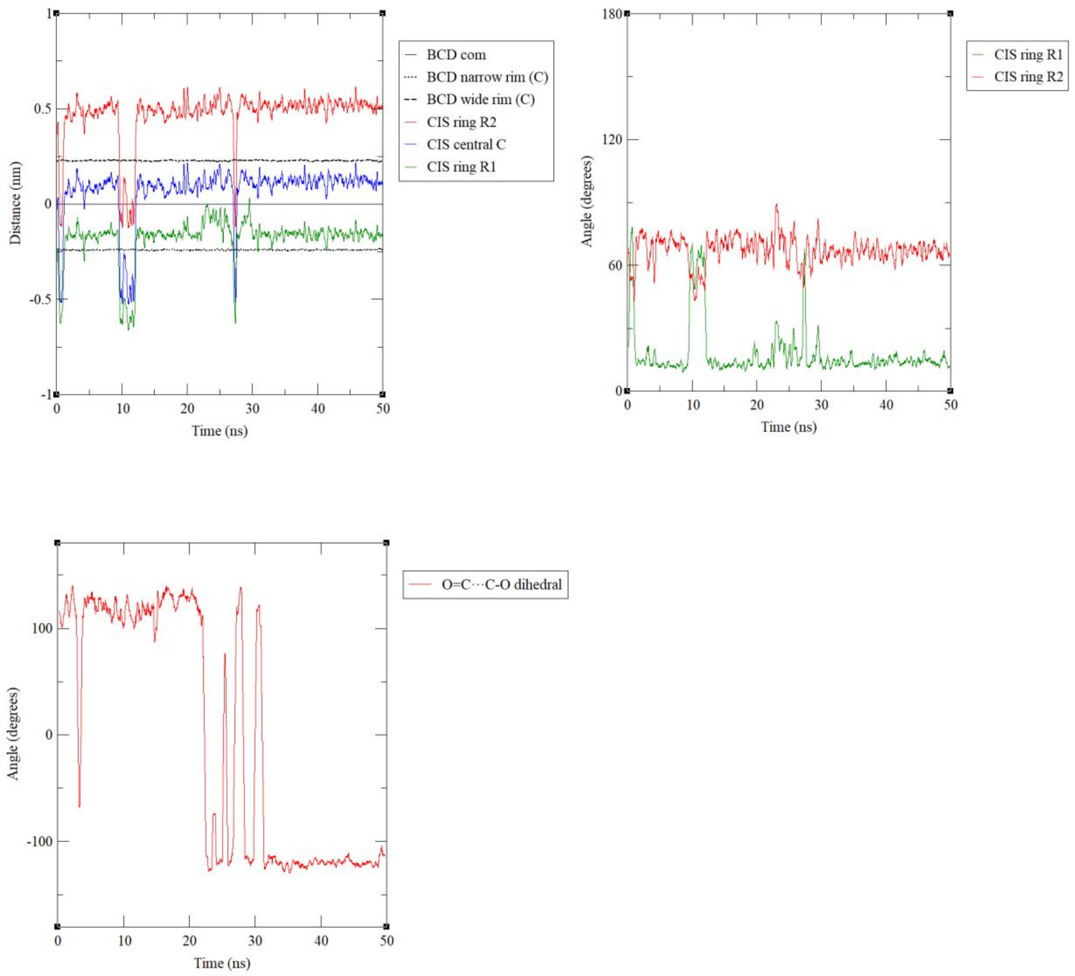

Figure S30 - (Up Left) Selected distances for the *cis*-chalcone arm included in β-CD taking the β-CD center of mass (com) as origin for measuring distances: the dashed black bottom line is the distance to the β-CD narrow rim formed by the carbon atoms of the hydroxy groups; the black dotted upper line is the distance to the β-CD wide rim formed by the oxygen atoms of the hydroxy groups; the red line is the distance to the com of the chalcone ring R2; the green line is the distance to the com of the chalcone ring R1; the blue line is the distance to the central carbon atom of the chalcone arm. (Up Right) Selected angles for the chalcone rings with the central axis of the β-CD. (Bottom) O=C···C—O dihedral angle in the cis-chalcone arm

Finally, MD simulations of systems containing the hemiketal branch were performed taking into account the two possible enantiomers that can arise from the closure of the cis form. Selected distances shown in Figure S31 indicate that the mobility of the hemiketal branch is small in the *β*-CD cavity. Even more, the analysis of the angles formed indicates that the R1 ring adopts an almost parallel disposition with the vertical axis of the cyclodextrin while the R2 ring, outside the cavity, is tilted around 50º.

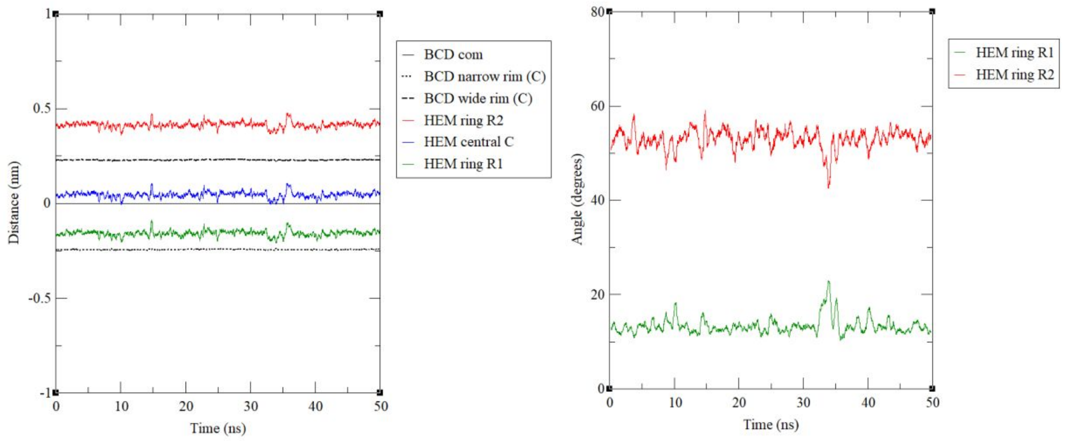

Figure S31 - (Left) Selected distances for the hemiketal arm included in *β*-CD taking the *β*-CD center of mass (com) as origin for measuring distances: the dashed black bottom line is the distance to the *β*-CD narrow rim formed by the carbon atoms of the hydroxy groups; the black dotted upper line is the distance to the *β*-CD wide rim formed by the oxygen atoms of the hydroxy groups; the red line is the distance of the hemiketal ring R2 to the com; the green line is the distance of the hemiketal ring R1to the com; the blue line is the distance to the central asymmetrical carbon atom of the hemiketal arm.

Spectra simulations

Theoretical circular dichroism spectra were obtained for selected molecular dynamics snapshots, keeping in mind that the large conformational variability of the chalcone arm prevents a complete theoretical reproduction of the experimental spectra. Thus, UV-Vis and ECD spectra for a representative conformation with the R2 ring clearly outside of the cyclodextrin cavity and with a *syn* disposition of C=O and OH group are presented in Figure S32 and both the theoretical UV-Vis and ECD spectra agree reasonably well with the experimental ones.

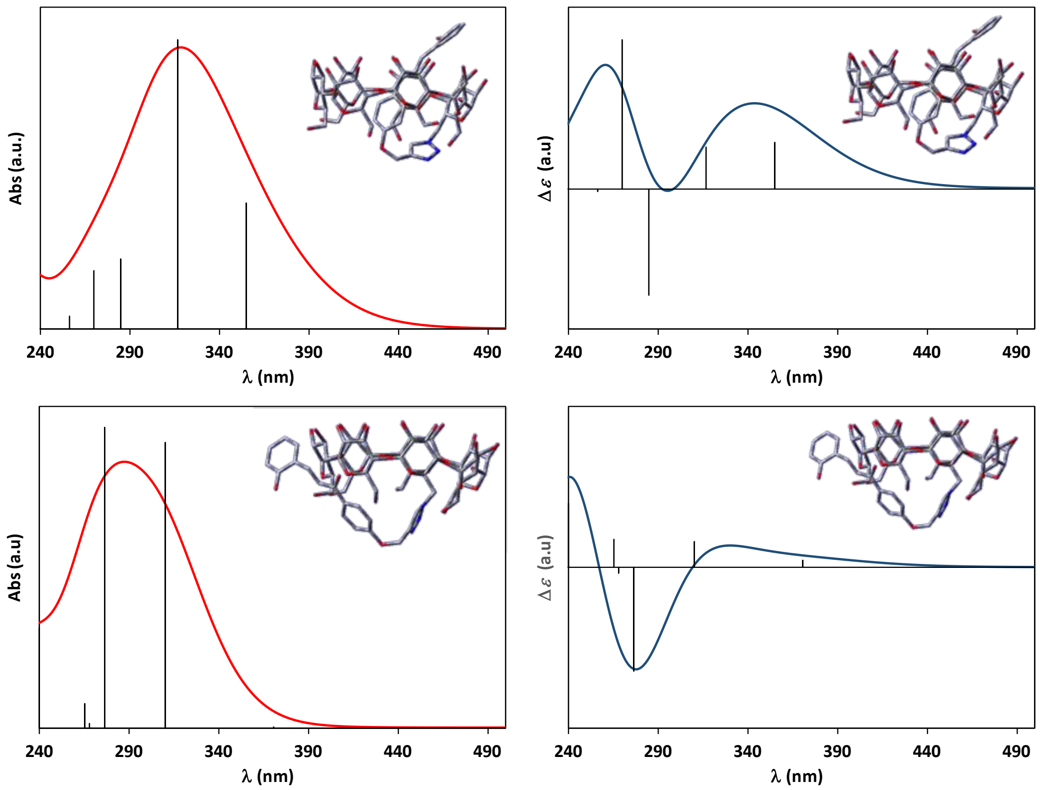

Figure S32 – (Up) Theoretical ECD and UV-vis spectra for a deeply buried conformation of the *trans*-chalcone arm. Bottom: Theoretical ECD and UV-vis spectra for a conformation of the *trans*-chalcone arm outside the *β*-CD cavity.

Electronic transitions within the 250-500 nm interval have been analyzed by means of natural transitions orbitals (NTOs)[3] to simplify its analysis because none of the

electronic transitions can be easily assigned to a single electronic transition between molecular orbitals. Employing Multiwfn software[4], we have also obtained the centroids of the NTOs, which can be used to analyze the charge transfer during electron excitation. According to the NTOs (see Figure S33), the lowest energy electronic transition (355 nm) is mainly located at the carbonyl-ene system. The second transition (316 nm) is associated to an electron density displacement from the inner R1 ring to the conjugated carbonyl-ene system. Remarkably, the geometrical centroids for the orbitals involved in these transitions are rather close to the central carbon atom of the chalcone and, therefore, close to the edge of the wider rim of the *β*-CD cavity. Following Kodaka rules,[5] these two transitions located inside the *β*-CD cavity show an enhanced positive CD in the experimental and theoretical spectra. The third electronic transition (285 nm) corresponds to an electron density displacement from the outer R2 ring of the chalcone branch towards the center of the molecule. Interestingly, the centroids of the natural orbitals involved indicate that a large part of this electron transition takes place mainly outside the *β*-CD cavity and, consequently, presents an induced negative CD signal. The highest energy transition (270 nm), corresponding to an electron density displacement from the inner R1 ring to the conjugated systems, takes place clearly inside the *β*-CD cavity and, therefore, presents an induced positive CD.

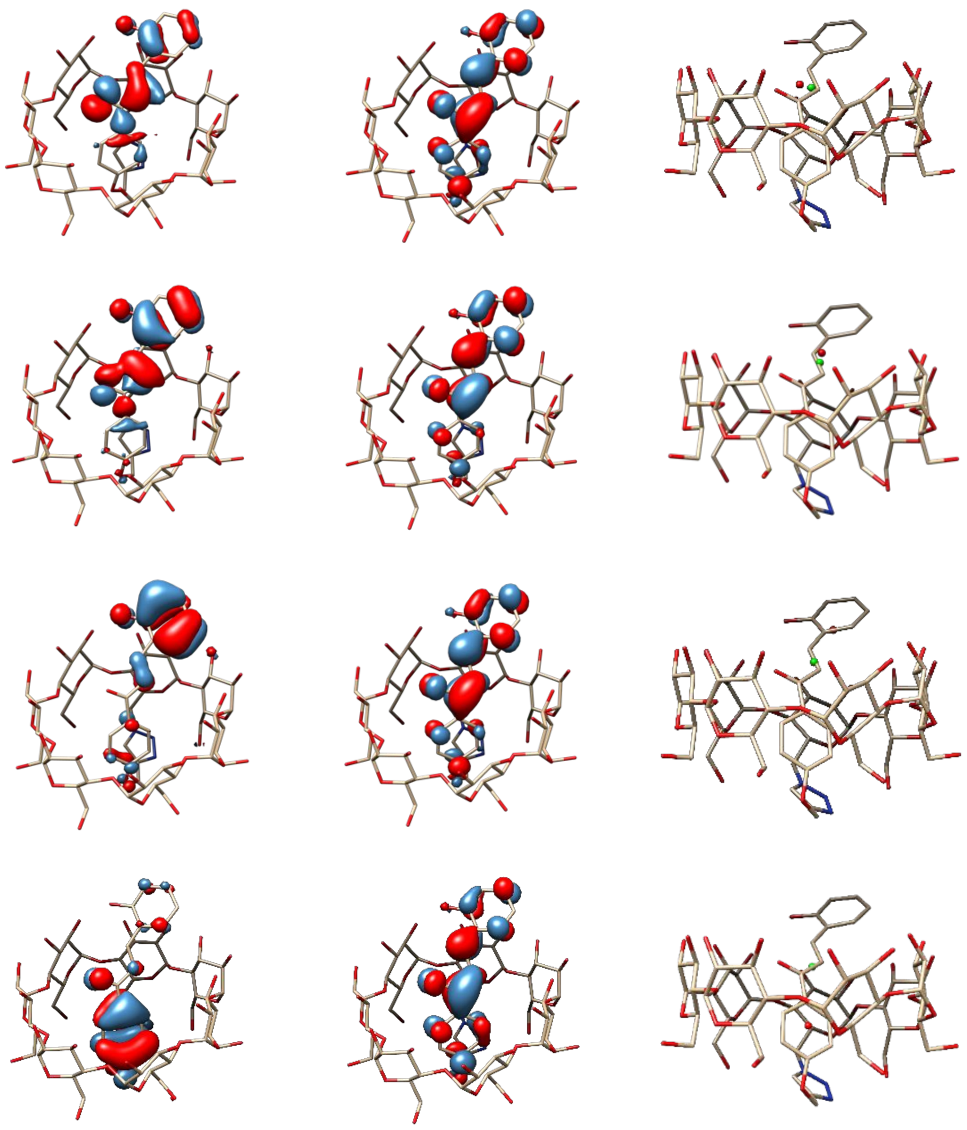

Figure S33 - Natural transition orbitals involved in electronic transitions between 250 and 400 nm. Centroids for the NTOs (red: occupied NTO/blue: virtual NTO)

We have also obtained the theoretical ECD of dispositions with the *trans*-chalcone arm outside the cyclodextrin and the analysis of their natural transition orbitals indicates that the nature of the electronic transitions is similar to that commented above. Therefore, the change in intensity observed in the ECD can be also explained through the Kodaka rules: every electronic transition associated with the ECD spectrum is now located outside the β-CD cavity and, as a consequence, the induced Cotton effect is reduced

significantly in the theoretical spectrum, which presents an almost zero signal at longer wavelengths and a negative band at around 280 nm which correlates appreciably with the experimental spectrum.

For the flavylium derivative included in the cyclodextrin cavity, the calculated ECD spectra were done for characteristic snapshots of MD simulations with both initial dispositions of the cation branch (see table S2). Interestingly, the results obtained for both orientations, shown in Figure S34, are almost mirror images and a combination of both ECD spectra agrees reasonably well with the absence of experimental ECD spectrum.

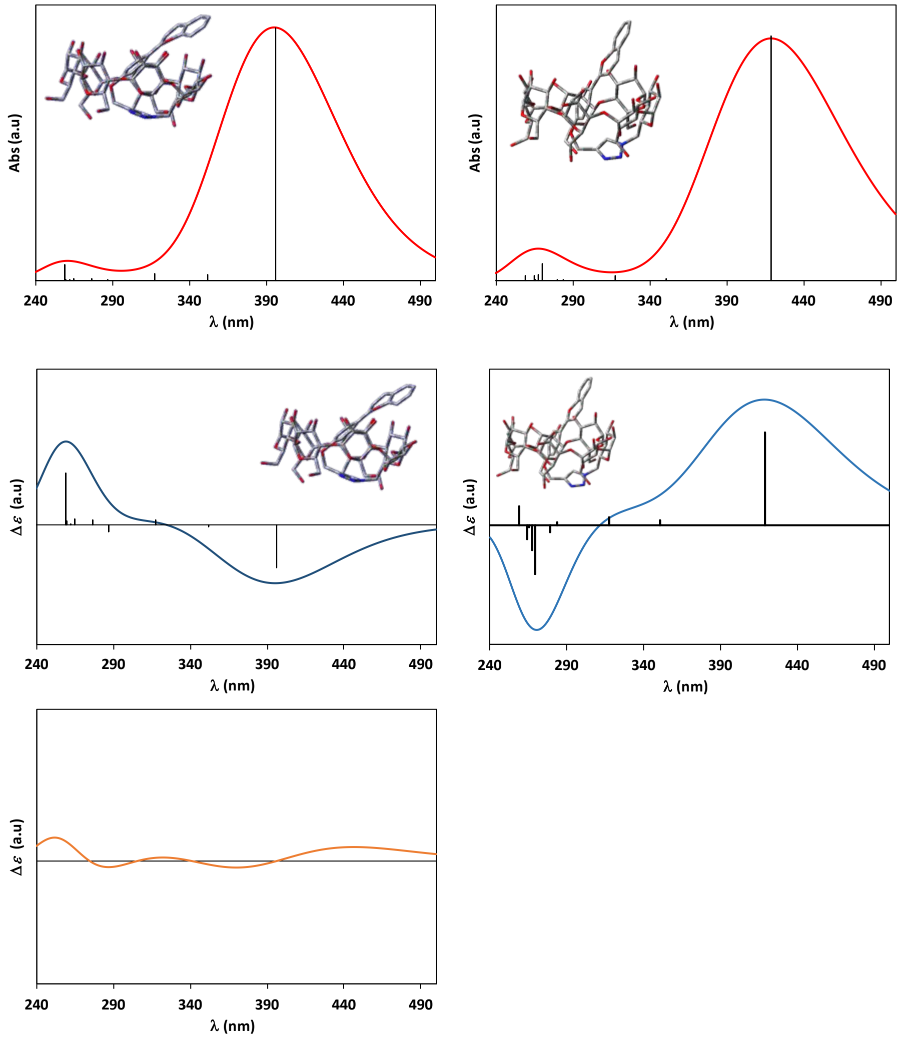

Figure S34 - Theoretical UV-vis and CD spectra for selected snapshots of MD simulations for the two different dispositions of the flavylium cation inside the *β*-CD cavity (note the different position of the oxygen atom in the aromatic system of the cation). The orange line is the CD spectrum corresponding to a 65:35 combination of both spectra.

For both forms, two main electronic transitions are responsible for the ECD profiles. The analysis of the NTOs associated to these transitions indicates, on one hand, that the band at higher wavelengths (around 400 nm) corresponds to the HOMO-LUMO transition within the resonant moiety of the flavylium cation. On the other hand, the band at shorter wavelengths (around 240 nm) corresponds to a strongly mixed electronic

transition involving mainly contributions from the rings of the flavylium arm but also with small contribution from orbitals from the *β*-CD unit, suggesting a certain amount of charge transfer between both parts of the molecule.

	Occupied	Virtual
402 nm	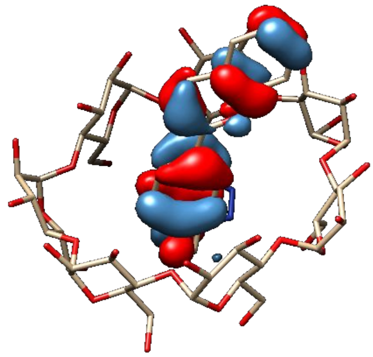	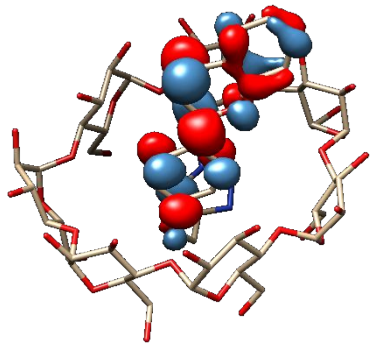
268 nm	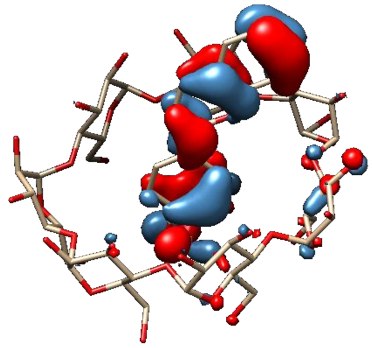	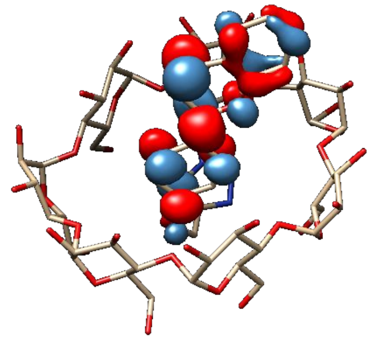

Figure S35 - Natural transition orbitals involved in electronic transitions between 250 and 500 nm.

Theoretical ECD spectra were also obtained for characteristic snapshots of MD simulations considering again both dispositions of the flavylium arm outside the *β*-CD unit (Fig S36). As observed previously for the flavylium unit residing inside the *β*-CD cavity, ECD spectra can be combined to agree with the experimental ECD spectrum. Finally, it must be also noted here that, according to the analysis of their natural transition orbitals, the nature of the electronic transitions for systems with the flavylium arm outside the cavity is similar to that already commented for systems within the *β*-CD cavity.

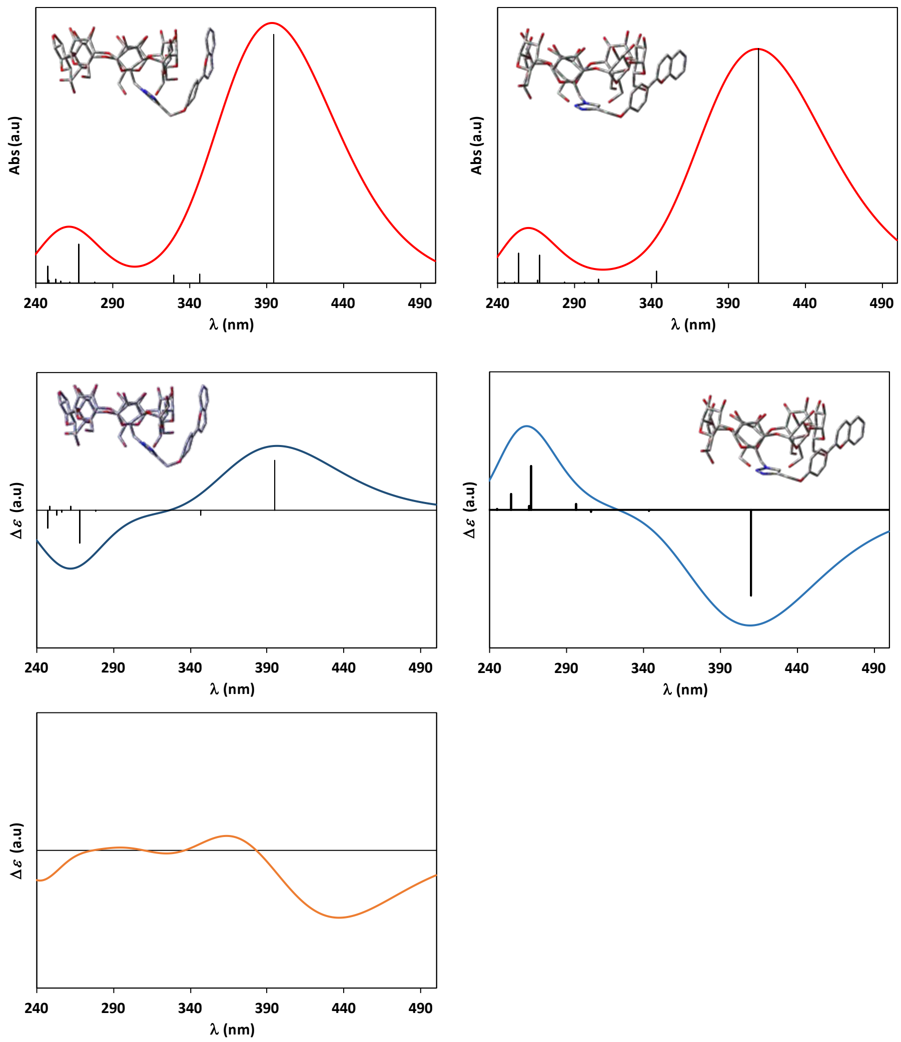

Figure S36 - Theoretical UV-vis and CD spectra for selected snapshots of MD simulations for the two different dispositions of the flavylium cation outside the β-CD cavity (note the different position of the oxygen atom in the aromatic system of the cation). The orange line is the CD spectrum corresponding to a 60-40 combination of both spectra

Finally, simulated ECD spectra for characteristic snapshots of systems containing the hemiketal arm inside and outside the cyclodextrine cavity were also obtained (see Figs S37-S38). Due to the presence of the central asymmetrical carbon atom, two hemiketal enantiomeric forms should be considered. The predicted ECD spectra for characteristic snapshots of both enantiomers are, as expected, almost mirror images. Following the

experimental results that suggest that a small amount of the cis form coexists with the hemiketal forms, we were able to obtain a good agreement between experiment and theory by combining the theoretical ECD spectra of characteristic conformations for both hemiketal enantiomers and both pro-*R* and pro-*S* forms of the *cis*-chalcone. Although the theoretical proportions employed do not match exactly the experimental ones, our theoretical results indicate that both the hemiketal and *cis*-chalcone forms must be considered to reproduce the experimental data.

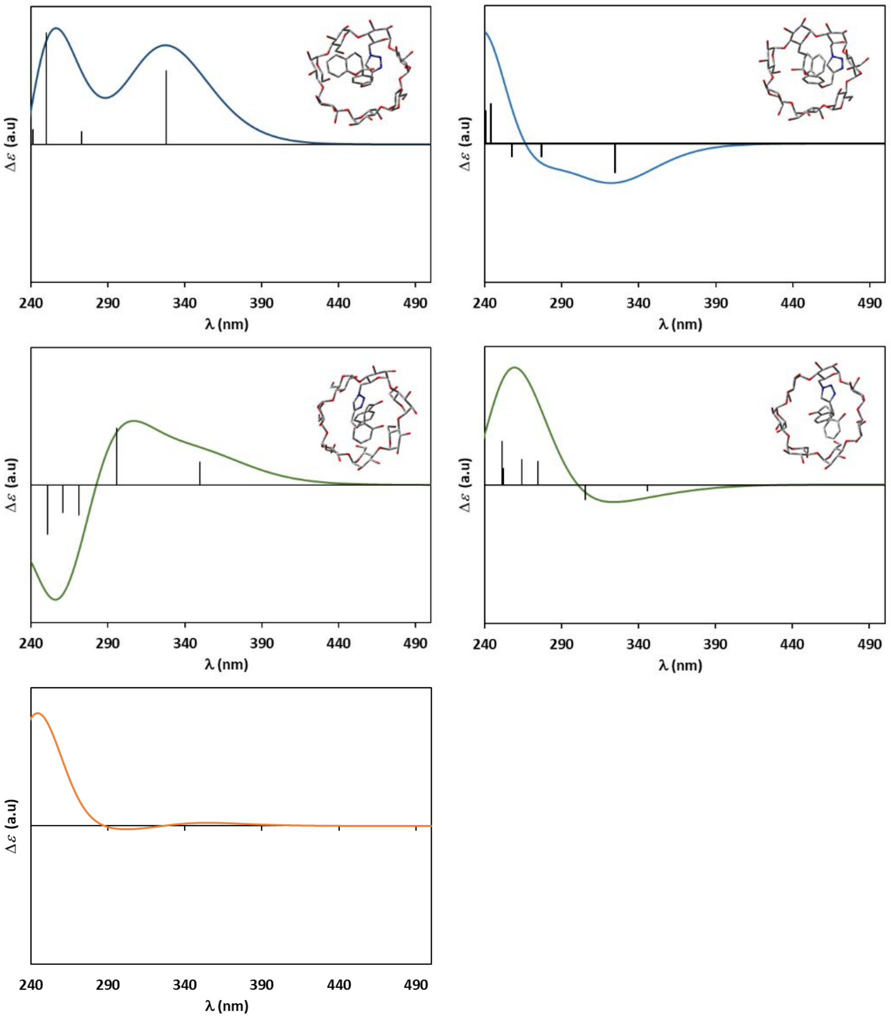

Figure S37 - (Upper line) Theoretical ECD spectra for selected snapshots of MD simulations for the *R* (left) and *S* (right) enantiomers of the hemiketal inside the *β*-CD cavity. (Medium line) Theoretical ECD spectra for selected snapshots of pro-*R* (left)

and pro-*S* (right) forms from MD simulations of the *cis*-chalcone inside the *β*-CD cavity (Bottom line) Theoretical CD spectrum corresponding to a 7:93 combination of the *cis*-chalcone/hemiketal spectra each one with contributions of 28:72 from *R*/pro-*R* and *S*/pro-*S* spectra.

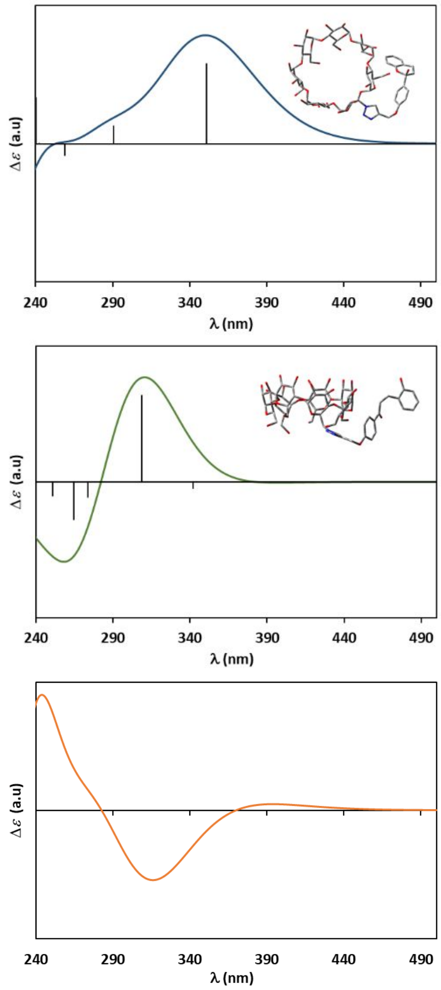

Figure S38 - (Upper line) Theoretical ECD spectra for selected snapshots of MD simulations for the *R* (right) and *S* (left) enantiomers of the hemiketal outside the *β*-CD cavity. (Medium line) Theoretical ECD spectra for selected snapshots of pro-*R* (right) and pro-*S* (left) forms from MD simulations of the *cis*-chalcone outside the *β*-CD cavity (Bottom line) Theoretical ECD spectrum corresponding to a 20:80 combination of the

cis/hemiketal spectra each one with contributions of 30:70 from *R*/pro-*R* and *S*/pro-*S* spectra.

4. Host-guest binding models

Considering the mechanism presented in Scheme 2 (see main text), the microscopic equilibrium expressions and the mass balance equations (equations 1-4) can be combined with equation 5 to obtain equation 6 which relates K_{app} with the microscopic binding constants K_{intra} and K_{AD}.

$$[1Ct]_0 = [1Ct^{IN}] + [1Ct^{OUT}] + [1Ct{:}AD] \quad (1)$$

$$[AD]_0 = [AD] + [1Ct{:}AD] \quad (2)$$

$$K_{intra} = \frac{[1Ct^{IN}]}{[1Ct^{OUT}]} \quad (3)$$

$$K_{AD} = \frac{[1Ct{:}AD]}{[1Ct^{OUT}][AD]} \quad (4)$$

$$K_{app} = \frac{[1Ct{:}AD]}{([1Ct^{IN}] + [1Ct^{OUT}])[AD]} \quad (5)$$

$$K_{app} = \frac{K_{AD}}{K_{intra} + 1} \quad (6)$$

Based on the mechanism shown in Scheme 3 (see main text), the following set of equations can be written to describe the binding of AD to **1-B** and **1-Cc**.

$$[1]_0 = [1Cc^{IN}] + [1Cc^{OUT}] + [1B^{IN}] + [1B^{OUT}] + [1B{:}AD] + [1Cc{:}AD] \tag{7}$$

$$[AD]_0 = [AD] + [1B{:}AD] + [1Cc{:}AD] \tag{8}$$

$$K_{tIN} = \frac{[1Cc^{IN}]}{[1B^{IN}]} \tag{9}$$

$$K_{tOUT} = \frac{[1Cc^{OUT}]}{[1B^{OUT}]} \tag{10}$$

$$K_{IntraCc} = \frac{[1Cc^{IN}]}{[1Cc^{OUT}]} \tag{11}$$

$$K_{IntraB} = \frac{[1B^{IN}]}{[1B^{OUT}]} \tag{12}$$

$$K_{AD:B} = K_{AD} = \frac{[1B{:}AD]}{[1B^{OUT}][AD]} \tag{13}$$

$$K_{AD:Cc} = K_{AD} = \frac{[1Cc{:}AD]}{[1Cc^{OUT}][AD]} \tag{14}$$

$$K_{tOUT} = K^{*}_{tOUT} = \frac{[1Cc{:}AD]}{[1B{:}AD]} \tag{15}$$

These equations can be combined to show that for this system the K_{app} is given by:

$$K_{app} = \frac{K_{AD}(1 + K_{tOUT})}{1 + K_{IntraB} + K_{IntraB}K_{tIN} + K_{tOUT}} \tag{16}$$

In addition, owing to the cyclic nature of the mechanism depicted in Scheme 3, it can be easily verified that:

$$K_{IntraCc} = K_{IntraB} \frac{K_{tIN}}{K_{tOUT}} \quad (17)$$

The approach described above can be extended to include the pH-dependent formation of the flavylium species (see Scheme 5 in the main text).

$$[1]_0 = [1Cc^{IN}] + [1Cc^{OUT}] + [1B^{IN}] + [1B^{OUT}] + [1AH^{+\,IN}] + [1AH^{+\,OUT}] + [1B{:}AD] + [1Cc{:}AD] + [1AH^+{:}AD] \quad (18)$$

$$[AD]_0 = [AD] + [1B{:}AD] + [1Cc{:}AD] + [1AH^+{:}AD] \quad (19)$$

$$K_{tIN} = \frac{[1Cc^{IN}]}{[1B^{IN}]} \quad (20)$$

$$K_{tOUT} = \frac{[1Cc^{OUT}]}{[1B^{OUT}]} \quad (21)$$

$$K_{IntraCc} = \frac{[1Cc^{IN}]}{[1Cc^{OUT}]} \quad (22)$$

$$K_{IntraB} = \frac{[1B^{IN}]}{[1B^{OUT}]} \quad (23)$$

$$K_{IntraAH+} = \frac{[1AH^{+\,IN}]}{[1AH^{+\,OUT}]} \quad (24)$$

$$K_{AD:AH+} = \frac{[1AH^+{:}AD]}{[1AH^{+\,OUT}][AD]} \quad (25)$$

$$K_{AD:B} = \frac{[1B{:}AD]}{[1B^{OUT}][AD]} \quad (26)$$

$$K_{AD:Cc} = \frac{[1Cc{:}AD]}{[1Cc^{OUT}][AD]} \tag{27}$$

$$K_{hIN} = \frac{[1B^{IN}][H^+]}{[1AH^{+\,IN}]} \tag{28}$$

$$K_{hOUT} = \frac{[1B^{OUT}][H^+]}{[1AH^{+\,OUT}]} \tag{29}$$

The above equation can be combined to show that the apparent K_a for the formation of the flavylium cation from the hemiketal and *cis*-chalcone species is given by:

$$K_a = K_{hOUT}\frac{1 + K_{IntraCc}K_{tOUT} + K_{tOUT} + K_{IntraB} + (1 + K_{tOUT})K_{AD}[AD]}{1 + K_{IntraAH+} + K_{AD}[AD]} \tag{30}$$

5. References

(1) Gago, S.; Basílio, N.; Quintas, A.; Pina, F. Effect of β-Cyclodextrin on the Multistate Species Distribution of 3-Methoxy-4′,7-Dihydroxyflavylium. Discrimination of the Two Hemiketal Enantiomers. *J. Agric. Food Chem.* **2017**, *65* (31), 6346–6358. https://doi.org/10.1021/acs.jafc.6b04892.

(2) Mendoza, J.; Basílio, N.; de Freitas, V.; Pina, F. New Procedure To Calculate All Equilibrium Constants in Flavylium Compounds: Application to the Copigmentation of Anthocyanins. *ACS Omega* **2019**, *4* (7), 12058–12070. https://doi.org/10.1021/acsomega.9b01066.

(3) Martin, R. L. Natural Transition Orbitals. *J. Chem. Phys.* **2003**, *118* (11), 4775–4777. https://doi.org/10.1063/1.1558471.

(4) Lu, T.; Chen, F. Multiwfn: A Multifunctional Wavefunction Analyzer. *J. Comput. Chem.* **2012**, *33* (5), 580–592. https://doi.org/10.1002/jcc.22885.

(5) Kodaka, M. A General Rule for Circular Dichroism Induced by a Chiral Macrocycle. *J. Am. Chem. Soc.* **1993**, *115* (9), 3702–3705. https://doi.org/10.1021/ja00062a040.
